# General control nonderepressible 1 interacts with cationic amino acid transporter 1 and affects *Aedes aegypti* fecundity

**DOI:** 10.1186/s13071-022-05461-x

**Published:** 2022-10-21

**Authors:** Matthew Pinch, Theodore Muka, Yashoda Kandel, Mahesh Lamsal, Nathan Martinez, Marialuisa Teixeira, Dmitri Y. Boudko, Immo A. Hansen

**Affiliations:** 1 Department of Biology, New Mexico State University, Las Cruces, NM USA; 2 ReCode Therapeutics, Dallas, TX USA

**Keywords:** *Aedes*, Fat body, Nutrient sensor, Amino acid transport, GCN1, Cationic amino acid transporter 1

## Abstract

**Background:**

The amino acid transporter protein cationic amino acid transporter 1 (CAT1) is part of the nutrient sensor in the fat body of mosquitoes. A member of the SLC7 family of cationic amino acid transporters, it is paramount for the detection of elevated amino acid levels in the mosquito hemolymph after a blood meal and the subsequent changes in gene expression in the fat body.

**Methods:**

We performed a re-annotation of *Aedes aegypti* cationic amino acid transporters (CATs) and selected the C-terminal tail of CAT1 to perform a yeast two-hybrid screen to identify putative interactors of this protein. One interesting interacting protein we identified was general control nonderepressible 1 (GCN1). We determined the expression pattern of GCN1 in several adult organs and structures using qRT-PCR and western blots. Finally, we knocked down GCN1 using double-stranded RNA and identified changes in downstream signaling intermediates and the effects of knockdown on vitellogenesis and fecundity.

**Results:**

In a screen for *Ae. aegypti* CAT1-interacting proteins we identified GCN1 as a putative interactor. GCN1 is highly expressed in the ovaries and fat body of the mosquito. We provide evidence that eukaryotic translation initiation factor 2 subunit alpha (eIF2α) phosphorylation changed during vitellogenesis and that RNA interference knockdown of GCN1 in whole mosquitoes reduced egg clutch sizes of treated mosquitoes relative to controls.

**Conclusions:**

*Aedes aegypti* CAT1 and GCN1 are likely interacting partners and GCN1 is likely necessary for proper egg development. Our data suggest that GCN1 is part of a nutrient sensor mechanism in various mosquito tissues involved in vitellogenesis.

**Graphical Abstract:**

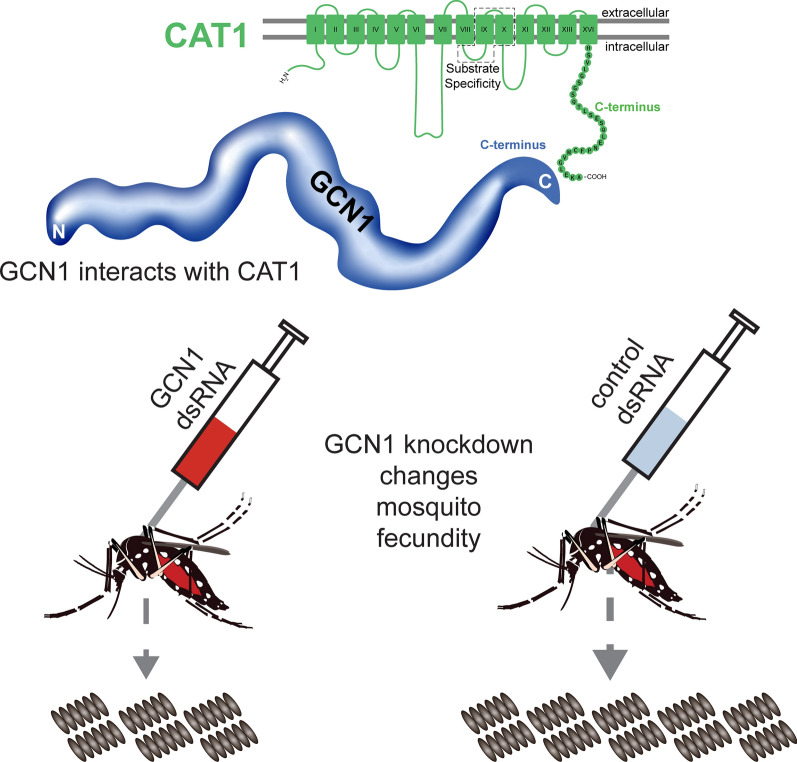

**Supplementary Information:**

The online version contains supplementary material available at 10.1186/s13071-022-05461-x.

## Background

The ability to sense nutrients and track changes in nutrient levels is of paramount importance for cell function and survival as well as maintenance of homeostasis in multicellular organisms. A variety of sensing and signaling pathways are used by eukaryotic cells to track specific nutrient concentrations and energy status [1]. Amino acids are the building blocks for all proteins that the cell produces to perform the functions necessary for survival and growth. Two conserved amino acid-sensing pathways have been identified in eukaryotes: (i) the mechanistic target of rapamycin (mTOR) complex 1 (mTORC1) signaling pathway; and (ii) the general amino acid control (GAAC) pathway, which was first described in yeast and is sometimes referred to in mammals as the amino acid response (AAR) pathway [2–4]. Both pathways regulate the synthesis of new proteins in response to cellular free amino acid content, with the mTORC1 pathway being known to integrate input from growth factors, energy status and nutrient availability to regulate cell growth [2–5].

Activation of mTORC1 is accomplished by recruitment of the complex to lysosomal membranes, where it is proposed to either sense free amino acid content of the lysosome or to interact with other cytoplasmic amino acid metabolic genes [2–4]. Once activated, the TOR kinase in mTORC1 phosphorylates another kinase, ribosomal protein S6 kinase beta-1 (p70-S6K), which in turn phosphorylates ribosomal protein S6, thereby “turning on” protein synthesis [3, 4, 6]. The central kinase in the GAAC pathway is general control nonderepressible (GCN) 2 (GCN2), which is activated in response to several cellular stressors, including low intracellular amino acid levels [7–11]. GCN2 senses these levels with the assistance of another protein, general control nonderepressible 1 (GCN1), which forms a complex with several proteins, including GCN2, general control nonderepressible 20 (GCN20) and ribosomes, allowing GCN2 to sense uncharged transfer RNAs (tRNAs) in the ribosomal A-site [12–15] and phosphorylate the eukaryotic translation initiation factor 2 subunit alpha (eIF2α) in response [12–18]. Phosphorylation of eIF2α causes a reduction in general translation and enhances translation of a subset of genes, including GCN4 (in mammals, activating transcription factor 4 [activating transcription factor 4]) which respond to amino acid starvation conditions [19–22]. Efforts are underway to understand how much crosstalk occurs between the mTORC1 and GCN2 signaling pathways [23–26].

The female yellow fever mosquito (*Aedes aegypti*) is an excellent model system to study amino acid signaling pathways that are active in a variety of organs and tissues [27, 28]. Female mosquitoes of this species require a blood meal to derive the nutrients necessary for egg production. Once blood is ingested into the midgut, nutrients, particularly amino acids from digested blood proteins, are secreted into the hemolymph from where they are taken up by the fat body and utilized to produce yolk precursor proteins (YPPs). YPPs are processed in the fat body, secreted into the hemolymph and absorbed by the developing oocytes via receptor-mediated endocytosis. The critical process of yolk formation in the eggs is called vitellogenesis [29].

Amino acid uptake by the fat body is essential for initiation of vitellogenesis, with cationic amino acids being absolutely required for the expression of the YPPs [30, 31]. The transport of cationic amino acids into the fat body during vitellogenesis is facilitated by proteins of the solute carrier family 7 (SLC7), including the heterodimeric amino acid transporters (HATs) and the cationic amino acid transporters (CATs) [32]. CAT proteins are 14-transmembrane domain transceptors, i.e. transporters that are thought to also act as receptors [33], whose substrate specificity is determined by an 80-amino acid region spanning from the fourth intracellular loop through the next two transmembrane domains (domains IX and X) [34]. CATs have been shown to play an important role in *Ae. aegypti* YPP synthesis during vitellogenesis, as demonstrated through RNA interference (RNAi) knockdown [32]. Five CAT proteins have been classified in *Ae. aegypti* [32], and the substrate specificity and transport dynamics of two of these have been characterized [30, 35, 36]. *Aedes aegypti* CAT 1 (AaCAT1) selectively transports histidine [36], while *Ae. aegypti* CAT 3 (AaCAT3) functions as a more general cationic amino acid transceptor, showing a slight preference for arginine [35]. The substrate profiles of the remaining AaCATs have not been classified.

Members of the CAT family have previously been shown to be part of the mTOR signaling pathway. RNAi knockdown of *Ae. aegypti* CAT 2 (AaCAT2) and AaCAT3 significantly reduced amounts of phosphorylated p70-S6K after stimulation of cultured fat bodies with amino acids [32]. While the link between CAT-mediated amino acid uptake and mTOR regulation of YPP gene expression in the fat body of *Ae. aegypti* has been firmly established, there is still no experimentally established signaling intermediary between CATs and the mTOR nutrient sensor.

Here we present evidence that AaCAT1 interacts with GCN1 in *Ae. aegypti*, and that GCN1 knockdown affects nutrient signaling and mosquito oocyte development.

## Methods

### Mosquito rearing

*Aedes aegypti* (Liverpool strain) mosquitoes were used for all experiments. Eggs were desiccated for at least 1 week prior to hatching in 13 × 20-in. pans, in deionized water at 27 °C. Larvae were fed Special Kitty dry cat food pellets (Walmart Stores Inc., Bentonville, AR, USA) every 3 days, and the water in each pan was changed every 5 days. Pupae were separated out into dishes and stored in Bug Dorm-1 insect rearing cages (30 × 30 × 30 cm; Bugdorm, Taichung, Taiwan) and allowed to emerge. Adult mosquitoes were maintained in their Bug Dorm cages under controlled conditions (27 °C, 80% humidity, 14:10-h light:dark cycle) and fed on 20% sucrose solutions ad libitum. Adult females at 5- to 7-day post-eclosion were used for all experiments.

### CAT annotation

*Aedes aegypti* CAT protein sequences were accessed from a previous publication [32] and from the National Center for Biotechnology Information (NCBI) protein database. *Drosophila melanogaster* protein sequences were accessed from FlyBase version FB2021_04 [37]. All *Ae. aegypti* and *D. melanogaster* protein sequences were aligned using Molecular Evolutionary Genetics Analysis (MEGA) software version 11 [38] using default settings, and a neighbor-joining tree was constructed using the default settings in MEGA 11. Nodes containing transcript variants or homologous sequences were collapsed, and relevant annotation information was manually added to the tree.

### Yeast two-hybrid assay

#### RNA isolation and messenger RNA enrichment

Wings, legs and heads of 100 blood-fed female adult mosquitos were removed and discarded. The remaining tissue was placed in 2-ml Eppendorf tubes with 0.5 ml of TRIzol® reagent (Thermo Fisher Scientific, Waltham, MA, USA). Tissue was homogenized using a VWR cordless motor (VWR, Avantor; Radnor, PA, USA; Cat. No. 4774-370) with disposable polybutylene terephthalate pestles (VWR, Avantor; Cat. No. 4774-358), and another 0.5 ml of TRIzol was added before the tubes were mixed by inversion. Total RNA was isolated and precipitated using the RNA Isolation with TRIzol:chloroform protocol. After phase separation, the aqueous phase containing total RNA was separated, and an equivalent volume of 100% ethanol was added to the aqueous phase and mixed. The samples were then purified using a Zymo RNA Clean and Concentrator 100 kit (Zymo Research, Irvine, CA, USA; Cat No. R1019) and in-column DNase treatment was performed to remove genomic DNA. Enrichment of the messenger RNA (mRNA) was performed using the purification of polyadenylated RNA protocol of Takara (Takara Bio USA Inc., San Jose, CA, USA). RNA concentrations were then measured on a Nanodrop™ 1000 spectrophotometer (Thermo Fisher Scientific).

#### Complementary DNA library construction

Complementary DNA (cDNA) libraries were constructed using the Make Your Own “Mate & Plate” Library System (Takara Bio USA Inc.) following the manufacturer’s instructions. Briefly, 2 µl of purified RNA sample, 1 µl of oligomerized deoxythymidine (oligo-dT) primer solution (CDSIII) and 1 µl of deionized water were incubated for 2 min at 72 °C and then cooled on ice for 2 min before being spun down briefly at 14,000 *g* for 10 s. To complete the reaction mixture, 2.0 µl 5× First-Strand Buffer Solution, 1.0 µl dithiothreitol (DTT; 100 mM), 1.0 µl Deoxyribonucleoside Triphosphated (NTP) Mix (10 mM) and 1.0 µl SMART MMLV Reverse Transcriptase were added to the solution. The resulting reaction mixture was incubated at 42 °C for 10 min before 1 µl of SMART III-modified oligo-dT was added. The solution was then mixed and incubated at 42 °C for 1 h. The reaction mixture was heated to 75 °C for 10 min to terminate first-strand synthesis and then cooled to room temperature. Finally, 1 µl of RNase H was added to the reaction mixture, which was incubated at 37 °C for 20 min.

The cDNA library was generated by long-Distance PCR amplification using the Advantage 2 Polymerase Mix (Takara Bio USA Inc.). The reaction mix consisted of 2 µl first-strand cDNA, 70 µl deionized water, 10 µl 10× Advantage 2 PCR buffer, 2 µl 50× dNTP mix, 2 µl 5’ PCR primer, 2 µl 3’ PCR primer, 10 µl 10× melting Solution and 2 µl 50× Advantage 2 polymerase Mix. Amplification was performed in an Eppendorf EPgradient MasterCycler (Eppendorf North America, Enfield, CT, USA) applying the following cycling parameters: 95 °C for 30 s; then 26 cycles of 95 °C for 10 s and 68 °C for 6 min; with a final cycle at 68 °C for 5 min.

#### Bait strain preparation

Membrane topology of the *Ae. aegypti* AaCAT1 protein (XP_021707900.1) was predicted using the ExPASy TMHMM v2.0 transmembrane prediction tool [39] and the Protter transmembrane prediction tool [40], resulting in identification of the N-terminus, C-terminus and intracellular regions. The TBLASTN function of the NCBI BLAST program [41] was used to identify the nucleotide sequence of the C-terminal intracellular sequence in *Ae. aegypti* codon usage. Primers flanking the C-terminal intracellular sequence were designed using NCBI Primer-BLAST [42], and cross-validated with NetPrimer (http://www.premierbiosoft.com/netprimer/). Adaptor sequences were added to the ends of the forward and reverse primers as specified in the Matchmaker Gold Yeast Two-Hybrid Kit (Takara Bio USA Inc.) to ensure insertion of the bait fragment into the pGBKT7 bait plasmid for use in the Matchmaker Gold Yeast Two-Hybrid Kit. Complete primer sequences (with adaptor regions denoted in italics) are as follows: forward—5′-*CATGGAGGCCGAATTC*CATTCGGTTCTTGGCTCCGGTAGT-3′; reverse—5′-*GCAGGTCGACGGATCC*TACGCCTTTTCGAGTCCTACCATG-3′.

PCR to generate the AaCAT1 bait fragment was performed using an Eppendorf EPgradient Mastercycler (Eppendorf North America) to generate the bait fragment for the two-hybrid screen. The bait primers were used in the reaction with the cDNA libraries generated in the previous step acting as a template. The In-Fusion HD Cloning Kit (Takara Bio USA Inc.) was used to clone the short and long fragments into the pGBKT7 plasmid vector. The Yeastmaker Yeast Transformation System 2 kit (Takara Bio USA Inc.) was then used to transform the bait plasmids into competent yeast cells of the Y2HGold strain following the manufacturer’s instructions.

#### Prey library construction

Competent yeast cells of the YI87 strain were prepared first according to the yeast transformation protocol in the Yeastmaker Yeast Transformation System 2 (Takara Bio USA Inc.). The Make Your Own “Mate & Plate” Library System (Takara Bio USA Inc.) was then used to complete the construction of the two-hybrid library following the manufacturer’s instructions.

#### Yeast two-hybrid screen (assessing bait and prey interactions)

The Matchmaker Gold Yeast Two-Hybrid System (Takara Bio USA Inc.) was used to mate the bait (Y2HGold) and prey (Y187) yeast strains following the manufacturer’s instructions. Mated yeast were then plated on synthetic defined (SD) growth medium lacking leucine and tryptophan (double drop out [DDO] media) supplemented with aureobasidin and X-alpha-Gal, a chromogenic substrate of the enzyme α-galactosidase.

To screen out potential false positives, blue colonies were picked and patched onto fresh DDO lacking adenine and histidine (quadruple drop out [QDO] media). Patched colonies were grown on QDO media for 3 days before another round of selection for blue colonies. Because yeast may carry multiple distinct plasmids, it was necessary to select out any non-interacting prey plasmids that may be harbored by blue colonies after growth on QDO media. To do this, blue colonies were picked from QDO media and patched onto fresh DDO media. Colonies were patched onto fresh DDO media twice, and only blue colonies at the end of this selection were kept for analysis of possible interactions.

#### Identifying protein interactors

After two rounds of selection on DDO, prey insert composition of the remaining blue colonies was checked by colony PCR using the Matchmaker Insert Check PCR Mix 2 (Takara Bio USA Inc.). PCR products were analyzed by electrophoresis on a 1% Tris-acetate ethylenediaminetetraacetic acid (EDTA) buffer (TAE) agarose/SYBR Safe gel. The appearance of more than one band was an indication of the presence of more than one prey plasmid in a cell. PCR products that produced single bands were purified and sent out to Molecular Cloning Laboratories (MCLAB) for sequencing. NCBI BLAST [43] was used to identify the *Ae. aegypti* gene sequences corresponding to the prey plasmid sequence data. A set of putative AaCAT1 interactors was selected from this dataset by removal of obvious false positives, such as colonies containing plasmids with no known mosquito insert, and interactors known to localize to the cytosol and plasma membrane were selected, as these two locations represent the regions where proteins are most likely to interact with AaCAT1.

### Mosquito protein isolations

Protein isolation from mosquitoes in the experiments described herein was performed as follows. Legs, heads and wings were removed from mosquitoes, and the structures were dissected as necessary for each assay described in the Results. Dissected organs and structures were homogenized using a VWR cordless motor (VWR, Avantor; Cat. No. 4774-370) with disposable polybutylene terephthalate pestles (VWR, Avantor; Cat. No. 4774-358) in 100 µl of lysis buffer (50 mM Tris, pH 7.4; 1% octylphenoxy poly(ethyleneoxy)ethanol, branched [IGEPAL]; 0.25% sodium deoxycholate; 150 mM NaCl; 1 mM EDTA) supplemented with 1 µl each of HALT™ protease inhibitor cocktail and HALT™ phosphatase inhibitor cocktail (both from Thermo Fisher Scientific). Homogenized samples were centrifuged for 10 min at 12,700 RPM and 4 °C to pellet the debris. The supernatant containing proteins was collected and the protein concentration was determined with a Pierce™ BCA Protein Assay Kit (Thermo Fisher Scientific) on a NanoDrop™ 1000 spectrophotometer (Thermo Fisher Scientific) using the Protein BCA program.

### Pull-down assays

A 6-histidine (6-His)-tagged AaCAT1 C-terminal bait fragment ([NH2]*HHHHHH*HSVLGSGSQTLSESQLENPFCMVGLEKA[COOH]) was custom synthesized (Pierce Biotechnology, Thermo Fisher Scientific) and used in conjunction with a Pierce™ Pull-Down PolyHis Protein:Protein Interaction Kit (Pierce Biotechnology, Thermo Scientific) with several modifications.

A set of protein samples were cross-linked to the 6-His tagged AaCAT1 bait prior to pull-down. Briefly, 1 mg disuccinimidyl sulfoxide (DSSO) crosslinker was dissolved in 51.5 µl dimethyl sulfoxide (DMSO) to make a 50 mM stock. Next, approximately 800 µg total protein isolate from fat bodies was mixed with 6-His-tagged CAT1 C-terminus at a concentration of 200 µg/ml in Tris-buffered saline (TBS) to generate two fat body samples. A 50-µl aliquot of dissolved crosslinker was added to one fat body sample, and 50 µl DMSO was added to a non-crosslinked fat body as control. Samples were incubated at 4 °C overnight to facilitate cross-linking prior to use in pull-down reactions. For protein pull-downs, 500 µl His-Pur cobalt resin per sample was centrifuged down for 2 min at 700 *g* and room temperature, and the supernatant was removed from the resin bed. Next, the resin was equilibrated in 500 µl of wash buffer (1:1 mix of TBS:Pierce Lysis buffer plus 5 mM imidazole) and centrifuged using the same settings as above. The wash buffer was removed, and the protein samples were added to the resin and mixed at room temperature with gentle rocking for 1 h to facilitate binding of cross-linked 6-His-tagged proteins to the His-Pur resin beads. After mixing, the samples were centrifuged as above, and the supernatant was collected and retained. The beads were washed 3 times with 200 µl of wash buffer each time, and after each wash the samples were centrifuged as described above, and the supernatant collected and retained each time. Finally, bound proteins were eluted in 100 µl of elution buffer (wash buffer containing 290 mM imidazole). After the beads were re-suspended in elution buffer, the samples were centrifuged as described above, and the supernatant was collected.

A second set of protein samples was pulled-down without cross-linking. Briefly, three 1.7-ml Eppendorf tubes (bait-only control, resin control and pull-down) were each filled with 200 µl of HisPure Cobalt Resin and equilibrated with five washes of 1.2 ml of wash solution (1:1 mix of TBS:Pierce Lysis Buffer plus 5 mM imidazole). Approximately 500 µg of bait protein was added to the bait only control and pull-down tubes, and an equivalent volume of wash solution was added to the resin control tube. All tubes were incubated at 4 °C with gentle rocking for 2 h to facilitate binding of 6-His tagged bait peptides to the HisPure Cobalt Resin. After incubation, the supernatant was removed, and the beads were washed once with 400 µl of wash solution. Approximately 2 mg of total protein lysate (see above for protein extraction protocol) was added to both the resin control and the pull-down tubes and an equivalent volume of wash solution was added to the bait-only control tube. All tubes were incubated at 4 °C with gentle rocking overnight to facilitate interaction between immobilized bait and prey proteins. The following day, the beads were transferred to the provided spin columns, and the flow-through from each sample was collected after centrifugation. Wash buffer (250 µl) containing 290 mM imidazole was added to each column, and the columns were incubated at room temperature for 5 min with gentle rocking followed by centrifugation to collect the eluted proteins.

### RNAi knockdown analysis

T7-conjugated double-stranded RNA (dsRNA) primers for *Ae. aegypti* GCN1 were designed from mRNA (GenBank reference sequence accession: XM_001651776.2), and green fluorescent protein (GFP) dsRNA primers were taken from a previous study [44] (Table 1). Total *Ae. aegypti* cDNA and a GFP-containing plasmid (3′ EGFP pXOON) were used as templates to produce T7-tagged dsDNA for use in dsRNA synthesis using the Megascript™ RNAi Kit (Thermo Fisher Scientific) following the manufacturer’s instructions. Adult female mosquitoes were injected by the oral route with 1 µl of approximately  1 µg/µl dsRNA intrathoracically using pulled glass capillaries and given 1 h to recover in Bug Dorm-1 insect rearing cages with 20% sucrose provided. After 1 h, any mosquitoes that had not recovered were discarded. Surviving injected mosquitoes were maintained using the rearing conditions described above until use in subsequent experiments. All injections were performed in females at 5–7 days post-eclosion to standardize mosquito ages with all other experiments performed.

**Table 1:** Double-stranded RNA primers

Primer	Sequence^a^	Fragment size (bp)
*gcn1* forward	*TAATACGACTCACTATAGGGAGA*TTCGCCTCATAAGAAGGTACGC	726
*gcn1* reverse	*TAATACGACTCACTATAGGGAGA*AATTGGGGTGGTTTGGATTTGC	
GFP forward^b^	*TAATACGACTCACTATAGGG*CGATGCCACCT	518
GFP reverse^b^	*TAATACGACTCACTATAGGG*CGGACTGGGTG	

*gcn1* General control nonderepressible 1, GFP green fluorescent protein

^a^T7 promoter regions upstream of gene-specific sequences are shown in italics

^b^GFP dsRNA primers were previously reported

### Western blot analyses

Total protein was isolated as described above from pools of various organs or structures for each assay. Total protein was mixed with an equivalent volume of 1:1 Laemmli buffer:beta-mercaptoethanol and heated at 95 °C for 10 min to denature the proteins. The samples were loaded into 7.5% sodium dodecyl sulphate-polyacrylamide gel electrophoresis (SDS-PAGE) gels (Bio-Rad Laboratories, Hercules, CA, USA) and ran at 100 V for 90 min (GCN1) or 60 min (all other proteins) using a PowerPac™ HC power source (Bio-Rad Laboratories) to separate proteins. Proteins were transferred from the gel to polyvinylidene difluoride (PVDF) membranes using pre-prepared transfer stacks (Bio-Rad Laboratories) in a Trans-Blot^®^ Turbo™ Transfer System (Bio-Rad Laboratories) using the standard 30 min pre-set transfer run. PVDF membranes were washed 3 times for 5 min in 1× TBS + 0.05% Tween-20 (TBST) and blocked in StartingBlock™ T20 (TBS) (Thermo Fisher Scientific) for 1 h at room temperature with gentle rocking. Membranes were incubated with primary antibody (Table 2) diluted in blocking buffer at 4 °C overnight with gentle shaking, followed by three washes for 5 min each in TBST and then by incubation with an appropriate secondary antibody (Table 2) diluted in blocking buffer for 1 h at room temperature with gentle shaking. The membranes were then washed 5 times for 5 min each time in TBST to remove any free secondary antibody and then incubated in Pierce 1-step TMB-Blotting Substrate Solution (Thermo Fisher Scientific) for colorimetric detection for up to 30 min at room temperature with gentle rocking. Alternatively, the blots were incubated in Li-Cor WesternSure® PREMIUM Chemiluminescent Substrate (Li-Cor, Lincoln, NE, USA) for 5 min at room temperature, following which the stained membranes were imaged using a C-DiGit Blot Scanner (Li-Cor) and Image Studio™ software (Li-Cor); pixel intensities were determined using this software for semi-quantitative densitometry measurements.

**Table 2:** Antibodies used for western blot assays

Antibody	Expected molecular weight (kDa)	Concentration	Manufacturer^a^
α-GCN1 primary—rabbit	293	1:1000	Abcam (ab86139)
α-eIF2α primary—mouse	36	1:250	Abcam (ab5369)
α-phospho-eIF2α primary—rabbit	36	1:250	Abcam (ab32157)
α-actin primary—mouse	43	1:500	MilliporeSigma (MAB1501)
α-mouse HRP secondary	N/A	1:1000 or 1:2000 (actin)	MilliporeSigma (12–349)
α-rabbit HRP secondary	N/A	1:1000	MilliporeSigma (12–348)

*eIF2α* Eukaryotic translation initiation factor 2 subunit alpha,* HRP* horseradish peroxidase

^a^Abcam, Cambridge, UK; MilliporeSigma, Burlington, MA, USA

### Quantitative reverse transcription PCR analysis

Mosquito organs and structures were dissected, and total RNA was extracted using a Qiagen RNeasy Mini Kit (Qiagen, Hilden, Germany). Ten organs and structures were pooled to generate individual samples, and a sample size of three was used for all quantitative reverse transcription PCR (qRT-PCR) experiments. RNA and potential genomic DNA concentrations were measured using a Qbit 2.0 (Invitrogen, Thermo Fisher Scientific), and RNA quality was confirmed using a NanoDrop 1000 spectrophotometer (Thermo Fisher Scientific). A 250-ng aliquot of RNA from each sample was reverse-transcribed using iScript™ Reverse Transcription Supermix for RT-qPCR (Bio-Rad Laboratories) to generate cDNA. To confirm the absence of genomic DNA contamination, non-reverse transcribed (noRT) samples were generated with iScript™ noRT Supermix (Bio-Rad Laboratories). Primers for *gcn1* were designed using PrimerBLAST [42] and evaluated with NetPrimer (Premier Biosoft, Palo Alto, CA, USA). Primers were designed to flank intron sequences to discriminate between mRNA and genomic DNA amplification products. Primers for the vitellogenin (*vg*), beta-actin (*β-actin*) and ribosomal protein S7 (*rps7*) genes have been published previously [45, 46] (see Table 3 for all primer sequences). All qRT-PCR primers were synthesized at the 10-nmol scale with standard desalting purification (Eurofins Genomics, Louisville, KY, USA).

**Table 3:** Quantitative reverse transcription PCR analysis primers

Primer	Sequence	Primer melting temperature (T_m_; °C)
*gcn1* forward	TCGGAGGAGCAAACCAATACG	60
*gcn1* reverse	TTCTTCAATTTCACGACGCAGG	
*vg* forward	ATGCACCGTCTGCCATC	60
*vg* reverse	GTTCGTAGTTGGAAAGCTCG	
*rps7* forward^a^	TCAGTGTACAAGAAGCTGACCGGA	60
*rps7* reverse^a^	TTCCGCGCGCGCTCACTTATTAGATT	
*β-actin* forward^a^	GACTACCTGATGAAGATCCTGAC	60
*β-actin* reverse^a^	GCACAGCTTCTCCTTAATGTCAC	

*β-actin* Beta-actin,* vg* vitellogenin,* rps7* ribosomal protein S7

^a^Internal reference genes

Because reference gene expression may differ between different organs or across different points during vitellogenesis, the RefFinder [47] tool was used to identify the most stably expressed reference gene for each qRT-PCR assay. For *gcn1* expression profiling in adult organs and structures, *rps7* was determined to be the appropriate reference, while *β-actin* was determined to be the appropriate reference for assaying *vg* transcription after RNAi treatment. All qRT-PCR runs were performed by diluting cDNA 1:1 in nuclease-free water and mixing the following components in clear 96-well plates (Genesee Scientific, El Cajon, CA, USA) on ice: 5 µl iTaq Universal SYBRgreen Supermix (Bio-Rad Laboratories), 1 µl of 1:1 mixed forward and reverse gene-specific primers, 1 µl diluted cDNA and 2 µl nuclease-free water. The plates were briefly spun down to collect all material at the bottom of the wells and sealed with ThermalSeal RTS™ clear plastic adhesive film (Excel Scientific Inc., Victorville, CA, USA). All qRT-PCR assays were run on a CFX96 Touch Real-Time PCR Detection System (Bio-Rad Laboratories) controlled by a computer using CFX Maestro software (Bio-Rad Laboratories) and the following cycling profile: an initial denaturation at 95 °C for 30 s; followed by 40 cycles of denaturation at 95 °C for 5s and a combined annealing/elongation at 60 °C for 30 s. Fluorescent measurement was performed after each elongation step. Melting curves from 65 °C to 95 °C in 0.5 °C increments with a 5-s hold at each step were performed immediately after each qRT-PCR. Primer pair amplification efficiency was determined using the raw fluorescence data in the Real-Time PCR Miner tool [48]. Average quantification cycle (*C*_q_) values from technical replicates of each sample were analyzed using the primer pair efficiencies determined by the Real-Time PCR Miner tool, and *gcn1* or *vg* *C*_q_ values were normalized to *rps7* or β*-actin* *C*_q_ values, respectively, to quantify changes in gene expression.

### Clutch size and hatch rate assay

Groups of female *Ae. aegypti* (5–7 days post-eclosion) were injected with either GCN1 dsRNA or GFP dsRNA as described in the above text. Five days post-injection (PI), surviving mosquitoes from both groups were blood-fed for 1 h using defibrinated bovine blood (HemoStat Laboratories, Dixon, CA, USA) warmed to 37 °C. Individual engorged mosquitoes were moved to egg laying chambers and maintained at the rearing conditions described above for 96 h to ensure adequate time for them to complete vitellogenesis and oviposition. Egg papers from each egg chamber were collected and desiccated for 96 h prior to counting for determination of the average clutch sizes from each treatment. After counting, eggs were desiccated for a minimum of an additional 72 h (total of at least 1 week of desiccation). To determine hatch rates, 12 egg papers from each treatment were randomly selected and placed in small dishes containing deionized water and a quarter pellet of Special Kitty cat food (Walmart Stores Inc.) and allowed to hatch. Larval counts from each clutch of eggs were compared to the number of eggs counted in each clutch to calculate hatch rates.

### Ovary morphology assay

Female mosquitoes 5–7 days post eclosion were injected with either GFP or GCN1 dsRNA as described above. At 5 days PI, female mosquitoes were given a blood meal and allowed to feed for 1 h. Directly prior to blood-feeding, a group of injected females was isolated for use as an unfed baseline sample. Groups of 10 blood-fed mosquitoes were sampled at 24, 48 and 72 h post-blood meal (PBM). Mosquitoes from each of the two injection treatments (GFP or GCN1 dsRNA) and four time points (unfed, 24 h PBM, 48 h PBM, 72 h PBM) were anesthetized on ice, and their ovaries were dissected. Both ovaries from each mosquito (*n* = 10) were measured and averaged to determine the average ovary length per mosquito for each treatment and time point. Five oocytes per mosquito (*n* = 10) were measured to determine average oocyte lengths from each mosquito for each treatment type and time point.

### Statistical analysis

Normality tests of the distributions of all data were performed using Shapiro–Wilk tests and QQ plots prior to selection of additional statistical tests. GCN1 qRT-PCR data from adult female organs and structures were analyzed using a one-way analysis of variance (ANOVA), and significant differences between samples was determined using a Tukey’s HSD post hoc test. Differences in phosphorylated eIF2α levels between GCN1 and GFP dsRNA-injected samples at different times PBM measured by western blot band densitometry were analyzed using a one-way ANOVA followed by a Tukey’s HSD post hoc test to identify any significant differences between treatments at each time point. Differences in *vg* expression between GCN1 and GFP dsRNA-injected samples measured by qRT-PCR were analyzed using unpaired t-tests at each time point. Differences in ovary and oocyte lengths of GCN1 and GFP dsRNA-injected mosquitoes were also analyzed using unpaired t-tests at each time point. Differences in egg number and hatch rates were analyzed using Mann–Whitney U-tests. Survival curves of dsRNA-injected mosquitoes were analyzed using a Log-rank Mantel-Cox test. *P*-values < 0.05 were to be considered statistically significant for all tests. All statistical analyses were performed using Prism 8 statistical analysis software (GraphPad Software, San Diego, CA, USA).

## Results

### Expansion of the *slimfast* gene family in mosquitoes

**Fig. 1:**
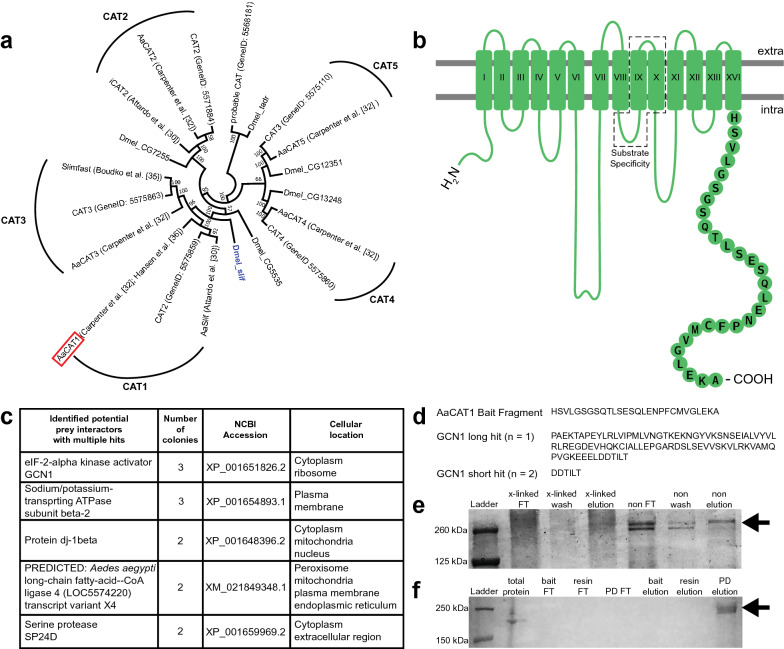
AaCAT1 phylogeny and interaction screen. **a** AaCAT1 and AaCAT3 represent an expansion of the *Drosophila* Slif CAT family. *Aedes aegypti* CAT protein sequences were downloaded from the NCBI protein database, and *Drosophila melanogaster* (Dmel) CAT protein sequences were accessed from FlyBase version FB2021_04 [37]. Protein sequences were aligned using MEGA11 [38], and a neighbor-joining tree was generated in MEGA11 from the multiple sequence alignment. Numbers next to each node represent bootstrap values based on 500 replications. Both AaCAT1 and AaCAT3 have previously been annotated as Slif in *Ae. aegypti*. Our current assembly shows that both transporters share homology with *D. melanogaster* Slif and represent an expansion of the Slif transporter family during *Ae. aegypti* evolution. The red box around AaCAT1 denotes its use in our protein interaction assay. All gene names not preceded by Dmel are *Ae. aegypti* CAT annotations. Brackets around gene clusters represent our suggested re-annotations of *Ae. aegypti* CATs based on this tree. **b** Predicted transmembrane structure of AaCAT1. The Protter web-based application [40] was used to illustrate the 14 transmembrane helices. The substrate specificity site was marked [34], and the C-terminal 28-amino acid fragment used for bait in the Y2H assay is indicated by circles containing their single-letter amino acid code. **c** Table containing the top five interacting proteins with at least two distinct hits determined by Y2H assay. **d** AaCAT1 bait fragment for pull-downs and GCN1 interacting fragments. **e** Pull-down analysis of AaCAT1-GCN1 interaction. The AaCAT1 C-terminal domain was incubated with fat body proteins and the resultant complexes captured on cobalt resin. Coomassie blue staining of crosslinked (x-linked) and non-crosslinked (non) pull-down reactions using a 6-His-tagged AaCAT1 bait fragment (same as in **d**), and fat body protein reveals an eluted band at the expected size for GCN1 interaction (marked by arrow). **f** GCN1 binds to the C-terminus of AaCAT1. Western blot of pull-down assay fractions confirms presence of GCN1 in the pull-down elution fraction (marked by arrow). bait, Bait-only control; FT, flow-through fraction; elution, eluted fraction; PD, pull-down; resin, resin control; wash, resin wash fraction; Y2H, yeast two-hybrid; for other terms, see Abbreviations

In previous publications, we have annotated the complete SLC7 gene family in *Ae. aegypti* [32] and identified the substrate affinities and transport activities of two of the five annotated CATs [35, 36]. Subsequent to these studies, new genome annotations have been published for *Ae. aegypti* [49, 50]. Because some of these automatic annotations assigned inconsistent numbers to particular CATs, we have chosen to re-annotate our previous CAT records [32] with the current annotations available in Entrez Gene, and with the current *D. melanogaster* CAT groups from FlyBase version FB2021_04 [37] for evolutionary context (see Additional file 1: Table S1). Multiple protein alignment in MEGA11 [38] revealed that both AaCAT1 and AaCAT3 cluster together closest to *D. melanogaster* Slimfast (Slif) (Fig. 1a), and thus likely represent a gene duplication from a common ancestor after the evolutionary split between flies and mosquitoes, 250 million years ago (MYA) [51]. Additionally, during this analysis, we identified two distinct gene entries for *Ae. aegypti* CAT2 and two distinct gene entries for *Ae. aegypti* CAT3 in the Entrez Gene database. Our neighbor-joining tree clustered one *Ae. aegypti* CAT2 (GeneID: 5575859) together with our AaCAT1 annotation [32], while the other *Ae. aegypti* CAT2 (GeneID: 5571884) clustered with our AaCAT2 annotation [32] (Fig. 1a). Additionally, one of the two *Ae. aegypti* CAT3 annotations (GeneID: 5575863) clustered with our AaCAT3 annotation [32], while the other *Ae. aegypti* CAT3 annotation (GeneID: 5575110) clustered with our *Ae. aegypti* cationic amino acid transporter 5 (AaCAT5) annotation [32] (Fig. 1a).

In silico analysis of the AaCAT1 amino acid sequence using the PyMOL molecular viewer tool (https://pymol.org/2/) and the TMHMM v2.0 transmembrane prediction tool [39] allowed us to predict the tertiary structure of the protein (Additional file 1: Figure S1a), as well as identify the transmembrane, intracellular and extracellular regions of the protein (Additional file 1: Figure S1b). We used these predicted domains, coupled with the CAT structure defined by Verrey and colleagues [34], to select the C-terminal end of AaCAT1 to use for yeast two-hybrid screening. We also determined that AaCAT1 likely localizes to the plasma membrane and lysosomes using the protein localization prediction tool, DeepLoc-1.0 [52] (Additional file 1: Figure S1c).

### Identification of AaCAT1-interacting proteins

Our yeast two-hybrid screen using the C-terminal tail of AaCAT1 as bait (Fig. 1b) yielded 207 colonies with the full complement of reporter gene activation on QDO high-stringency selective media (Additional file 2: Dataset). Prey plasmids were isolated from these colonies and sequenced. After removal of both obvious and likely false positives based on prey plasmid sequence analysis and known tissue and subcellular localizations of prey proteins, 13 likely protein interactors remained (Additional file 1: Table S2). Of these, five interactors were detected in at least two distinct colonies (Fig. 1c). The eIF2-alpha kinase activator, GCN1 was the most interesting of these, as we identified three distinct colonies derived from two separate clones containing plasmids encoding interacting peptides from the C-terminal end of GCN1 (Fig. 1c, d), and because GCN1 has previously been demonstrated to participate in GAAC regulation of translation in other eukaryotes [7, 9, 10, 14, 53].

### Pull-down confirms AaCAT1-GCN1 interaction

We confirmed the interaction between AaCAT1 and GCN1 by designing a 6-His-tagged peptide corresponding to the same 28 amino acid-long sequence of AaCAT1 C-terminus used for our yeast two-hybrid bait. Coomassie blue staining revealed the retention of a band > 260 kDa in the elution fraction of both crosslinked and non-crosslinked pull-down assays (Fig. 1e). Western blotting of non-crosslinked pull-down assay samples with an anti-GCN1 antibody confirmed the presence of GCN1 in the pull-down elution fraction (Fig. 1f).

### GCN1 expression in different mosquito organs

**Fig. 2:**
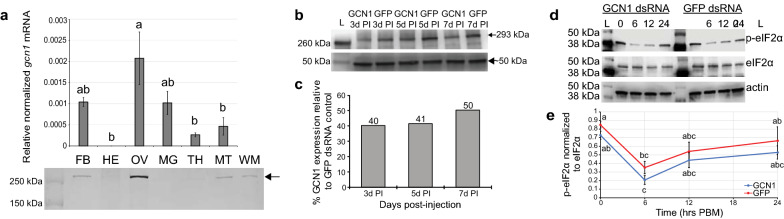
GCN1 is expressed in reproduction-associated tissues, and its signaling activity is reduced after RNAi-mediated knockdown. **a** qRT-PCR profiling of GCN1 expression in adult tissues (top) and western blotting of GCN1 protein expression (bottom) revealed expression of GCN1 in ovaries, fat body and Malpighian tubules. A one-way analysis of variance (ANOVA) followed by Tukey’s HSD post hoc test was used to determine significant differences (*P* < 0.05) in *gcn1* transcript expression. A 15-µg aliquot of protein from each sample was loaded for western blot detection. **b** Western blot of GCN1 expression after dsRNA injection. **c** Percentage GCN1 expression relative to GFP dsRNA treatment shows similar decrease in GCN1 expression at 3- and 5-days post-injection (PI), with an increase in relative GCN1 expression beginning by 7 days PI. Pixel intensity from GCN1 bands in **2b** (293 kDa, arrow) was standardized using the pixel intensity of the bright approximately 50-kDa band (arrowhead), which had similar intensities across all samples. Total protein was extracted from pools of 10 whole mosquitoes for each sample (*n* = 1) and 15 µg of protein from each sample was analyzed. **d** Representative western blots of phospho-eIF2α (p-eIF2α), eIF2α, and actin from three pools of five female abdomens (*n* = 3), with midguts removed, after injection with either GCN1 dsRNA or green fluorescent protein (GFP) dsRNA. Injected mosquitoes were sampled at different time points post-blood meal (PBM): 0 (unfed), 6, 12 or 24 h PBM. A 10-µg aliquot of total protein from each sample was loaded. **e** Pixel intensities of p-eIF2α bands from GCN1-injected and GFP-injected mosquitoes in blot from **2d** was normalized to pixel intensity of eIF2α bands from **2d** to plot change in p-eIF2α band intensity at different time points PBM. Letters represent statistical significance groups (*P* < 0.05) as determined by analysis with a one-way ANOVA followed by Tukey’s HSD post hoc test for significant differences between treatment groups and time points. FB, fat body; HE, head; OV, ovary; MG, midgut; TH, thorax; MT, Malpighian tubule; WM, whole mosquito; for other terms, see Abbreviations

GCN1 transcripts in adult female organs as detected by qRT-PCR were highest in organs associated with blood meal digestion and vitellogenesis. Relative GCN1 mRNA levels were significantly higher in ovaries than in heads, thoraxes and Malpighian tubules (ovaries/heads: ANOVA, *F*_*(5,12)*_ = 6.391, *P* = 0.0033; ovaries/thoraxes: ANOVA, *F*_*(5,12)*_ = 6.391, *P* = 0.0094; ovaries/Malpighian tubules: ANOVA, *F*_*(5,12)*_ = 6.391, *P* = 0.0212) (Fig. 2a). Fat bodies and midguts, which are other organs necessary for vitellogenesis, expressed GCN1 mRNA at levels not significantly different from those in the ovaries (ovaries/fat bodies: ANOVA, *F*_*(5,12)*_ = 6.391, *P* = 0.2028; ovaries/midguts: ANOVA, *F*_*(5,12)*_ = 6.391, *P* = 0.1877). Western blot analysis of adult female organs revealed GCN1 protein levels to be highest in ovaries. We also detected GCN1 protein in fat body and Malpighian tubules but interestingly not in midguts. Finally, we detected low levels of GCN1 protein in both head and thoraxes. This expression pattern matches that of many of the organs in which AaCAT1 has previously been detected [36], with the major tissues responsible for vitellogenesis containing detectible levels of both AaCAT1 and GCN1. In silico analysis of GCN1 subcellular localization using DeepLoc-1.0 [52] shows GCN1 most likely localizes to the cytosol but may also localize to the nucleus (Additional file 1: Figure S2a) and contains multiple putative nuclear localization sequences, as predicted using the cNLS Mapper tool [54] (Additional file 1: Figure S2b, c).

### Optimization of GCN1 knockdown

To determine an optimal time frame for RNAi manipulations, we injected small numbers of female mosquitoes with 1 µg of either GCN1 dsRNA or GFP dsRNA and isolated total protein from pools of 10 mosquitoes (*n* = 1) at 3-, 5- and 7-days PI. We performed western blots with an antibody against mammalian GCN1 to probe for GCN1 protein expression in all samples (Fig. 2b). Western blots with this primary antibody showed a GCN1 band at approximately 290 kDa and an additional band at approximately 50 kDa with consistent intensity in all samples (see Additional file 1: Figure S4d). We used band densitometry to semi-quantify pixel intensities of GCN1 bands (293 kDa) and normalized them to the 50 kDa band which we used as a quasi-internal loading control. Percentage reduction in GCN1 band intensity of GCN1 RNAi samples relative to GFP RNAi samples at each time point were graphed, and we observed an approximately 60% reduction in GCN1 protein levels in both 3- and 5-day PI samples (Fig. 2c).

### GCN1 knockdown does not cause mortality

To elucidate the role of GCN1 in *Ae. aegypti* vitellogenesis, we designed and synthesized anti-GCN1 dsRNA to knock down GCN1 gene expression. Because GCN1 has been shown to be involved in nutrient signaling and starvation response in other organisms [9–12, 14, 15, 53], we wanted to understand whether GCN1 knockdown would cause rapid lethality and be detrimental for future knockdown experiments in *Ae. aegypti* mosquitoes. To assess the lethality of GCN1 knockdown, we injected approximately 200 female mosquitoes with either 1 µg GCN1 dsRNA (*n* = 199) or 1 µg GFP dsRNA (*n* = 205) as an injection control and measured percentage mortality over 5 days PI. Both groups, i.e. the knockdown and GFP-control groups, had > 80% survival 5 days PI (Additional file 1: Figure S3a). Survival rates of GCN1 dsRNA-injected mosquitoes were not significantly different than those of GFP dsRNA-injected controls at any time point measured (log-rank Mantel-Cox test,* χ*^2^ = 0.02547, *df* = 1, *P* = 0.8732) (Additional file 1: Figure S3a).

### eIF2α phosphorylation changes significantly early in vitellogenesis but is not significantly decreased after GCN1 knockdown

It is known that GCN1 regulates GCN2, which in turn phosphorylates eIF2α and regulates protein translation in yeast [12, 14]. To test if GCN1 dsRNA injections led to functional GCN1 activity knockdown, we performed western blots on three pools of total protein isolated from abdomens of five mosquitoes at different time points PBM (*n* = 3 at all time points) with the midguts removed to avoid blood contamination (Fig. 2d; Additional file 1: Fig. S4e–g). We observed a significant decrease in eIF2α phosphorylation in both treatment groups at 6 h PBM relative to unfed mosquitoes in each group (GCN1 unfed-GCN1 6 h PBM: ANOVA, *F*_*(7,16)*_ = 5.562, *P* = 0.0128; GFP unfed-GFP 6 h PBM: ANOVA, *F*_*(7,16)*_ = 5.562, *P* = 0.0183) (Fig. 2e). This indicates that the GAAC pathway is an active nutrient sensing switch in female *Ae. aegypti* as they metabolize a blood meal during vitellogenesis.

When comparing eIF2α phosphorylation between treatment groups, we observed decreased levels of phosphorylated eIF2α (Fig. 2d, top panel) in mosquitoes injected with GCN1 dsRNA relative to mosquitoes injected with GFP dsRNA at all time points (Fig. 2e); however, this decrease was not significant at any time point measured (unfed: ANOVA, *F*_*(7,16)*_ = 5.562, *P* = 0.9732; 6 h PBM: ANOVA, *F*_*(7,16)*_ = 5.562, *P* = 0.9340; 12 h PBM: ANOVA, *F*_*(7,16)*_ = 5.562, *P* = 0.9890; 24 h PBM ANOVA, *F*_*(7,16)*_ = 5.562, *P* = 0.9506). Levels of total eIF2α and actin, used as loading controls were similar in both treatments (Fig. 2d, middle and bottom panels), which gave us confidence that the decreased eIF2α phosphorylation we observed in the GCN1 dsRNA-injected mosquitoes was due to our knockdown treatment.

### GCN1 knockdown does not affect *vitellogenin* expression

We blood-fed mosquitoes injected with both GCN1 dsRNA and GFP dsRNA 5 days PI, and then sampled groups of mosquitoes at different time points PBM for qRT-PCR analysis of *vg* expression. We observed a 2000-fold increase in *vg* mRNA 24 h PBM relative to the unfed state in GFP-injected mosquitoes (Additional file 1: Figure S3b). Interestingly, we did not observe a reduction in *vg* expression in GCN1-injected mosquitoes relative to GFP-injected mosquitoes, as *vg* mRNA increased almost 3800-fold in GCN1 dsRNA-injected mosquitoes (Additional file 1: Figure S3b). The expression of *vg* mRNA was not significantly different at any time point measured (unfed: unpaired t-test, *t*_(4)_ = 1.299, *P* = 0.2637; 6 h PBM: unpaired t-test, *t*_(4)_ = 0.2851, *P* = 0.7897; 12 h PBM: unpaired t-test, *t*_(4)_ = 1.275, *P* = 0.2713; 24 h PBM: unpaired t-test, *t*_(4)_ = 0.1612, *P* = 0.8797).

### GCN1 knockdown affects mosquito fecundity

**Fig. 3:**
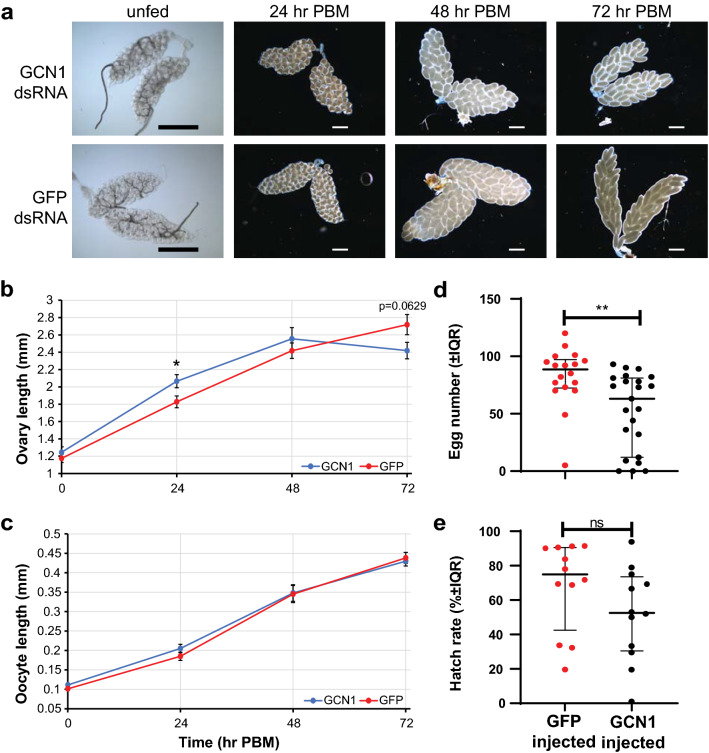
GCN1 knockdown affects mosquito fecundity. **a** Representative ovaries imaged from mosquitoes injected with either GCN1 dsRNA or GFP dsRNA at stated times PBM. Scale bars in each image: 500 µm. **b**, **c** Ovary lengths (**b**) and lengths of five representative oocytes (**c**) were measured from 10 GCN1 dsRNA-injected (*n* = 10) or 10 GFP dsRNA-injected (*n* = 10) mosquitoes sampled at 0 h PBM (unfed), 24 h PBM, 48 h PBM or 72 h PBM. Data are presented as the average length of the 10 ovary pairs (**b**) or oocytes (**c**) ± standard error of the mean. Paired t-tests were used to determine statistical significance of differences between GCN1 dsRNA-injected and GFP dsRNA-injected ovary and oocyte lengths at each time point. **d** Egg numbers of GFP dsRNA-injected (*n* = 18) and GCN1 dsRNA-injected (*n* = 23) mosquitoes. Individual dsRNA-injected females were blood-fed 5 days PI and separated into individual egg laying chambers. Egg paper with deposited eggs were collected 96 h PBM and desiccated for 48 h prior to counting. Each point represents the number of eggs laid by a single mosquito. Lines and whiskers represent median egg number ± interquartile range (IQR). **e** Hatch rates of 12 (*n* = 12) randomly selected clutches from mosquitoes injected in part **c**. Lines and whiskers represent median percent of eggs hatched per clutch ± IQR. Statistical significance (*P* < 0.05) of egg number and hatch rate data was determined with Mann-Whitney U-tests using GraphPad Prism 8 software

To determine the effects of GCN1 knockdown on mosquito fecundity, we injected female mosquitoes with GCN1 or GFP dsRNA and then dissected ovaries from groups of unfed (0 h PBM) and blood-fed females at 24, 48, and 72 h PBM (Fig. 3a). Ovary lengths grew at a greater rate in GCN1-injected mosquitoes through the first 48 h PBM, with lengths being significantly different at 24 h PBM (24 h PBM: un-paired t-test, *t*_(18)_ = 2.333, *P* = 0.0314; 48 h PBM: un-paired t-test, *t*_(18)_ = 0.8753, *P* = 0.3930). However, GCN1-injected mosquito ovaries ceased growing from 48 to 72 h PBM, when they were overtaken in length by ovaries from GFP-injected females, although the difference was not significant (unpaired t-test, *t*_(18)_ = 1.982, *P* = 0.0629) (Fig. 3b). Oocyte lengths were not different at any time point measured (unfed: unpaired t-test, *t*_(18)_ = 1.571, *P* = 0.1335; 24 h PBM: unpaired t-test, *t*_(18)_ = 1.339, *P* = 0.1972; 48 h PBM: unpaired t-test, *t*_(18)_ = 0.09389, *P* = 0.9262; 72 h PBM: unpaired t-test, *t*_(18)_ = 0.4433, *P* = 0.6628) (Fig. 3c).

We measured clutch sizes from mosquitoes injected with both GCN1 dsRNA (*n* = 23) and GFP dsRNA (*n* = 18) mosquitoes. GCN1 knockdown caused a significant (Mann–Whitney U-test, *U*_(39)_ = 92.50, *Z* = − 3.01, *P* = 0.0021) reduction in clutch size compared to GFP-injected controls, with a median of 63 eggs per clutch in the GCN1 treatment group compared to a median of 88.5 eggs per clutch in the GFP control group (Fig. 3d). We also measured hatch rates from 12 randomly selected clutches of the GCN1 knockdown- and GFP dsRNA-injected control mosquitoes. Clutches laid by GCN1-knockdown mosquitoes had a median percentage hatch rate of 53% and control clutches had a median percentage hatch rate of 75%. This difference was not statistically significant different (Mann–Whitney U-test, *U*_(22)_ = 44, *Z* = − 1.62, *P* = 0.1135) (Fig. 3e).

## Discussion

In this study, we identified a new set of signaling proteins that are likely part of the nutrient sensor in the fat body, ovaries and other tissues of the mosquito [6, 30, 31, 35, 36, 45, 55, 56]. We discovered GCN1 as a putative interaction partner of the C-terminus of a CAT and showed that GCN1 knockdown affects amino acid-induced nutrient signaling and mosquito fecundity.

We started our study by re-annotating the five mosquito CAT proteins of the SLC7-type amino acid transporter gene family [32, 34, 57]. The NCBI gene database contains two distinct entries for *Ae. aegypti* CAT2 and CAT3 (AaCAT2, AaCAT3), and no entries for *Ae. aegypti* CAT1 or CAT5 (AaCAT1, AaCAT5). Our analysis (Fig. 1a) supports re-annotation of the CAT2 (GeneID: 5575859) gene entry as CAT1, and the CAT3 (GeneID: 5575110) gene entry as CAT5. Both AaCAT1 and AaCAT3 clustered together with *D. melanogaster* Slif (Fig. 1a), indicating that AaCAT1 and AaCAT3 represent an expansion of the Slif CAT family during the evolutionary history of *Ae. aegypti*. In earlier studies we showed that both of these closely related proteins are amino acid transporters but with completely different substrate specificities. While AaCAT3 has broad substrate specificity and transports not only all four types of cationic amino acids but also several uncharged ones [35], AaCAT1 is a uniquely histidine-specific transporter that is part of the fat body nutrient sensor [36]. Because AaCAT1 knockdown has been shown to interfere with fat body nutrient signaling, placing it high upstream within the target of rapamycin (TOR) signaling pathway [30], we chose the AaCAT1 C-terminus as bait to identify interacting cytoplasmic proteins.

Our yeast two-hybrid screen of the AaCAT1 C-terminus produced 33 protein hits we deemed as plausible interactors of AaCAT1. Many of these might be true interactors of AaCAT1 and will be candidates for further studies. Overall, the collection of strongly interacting proteins we identified (Fig. 1c) hint at the possibility that AaCAT1 is part of a complex designed to manage the charge distribution associated with transporting charged amino acids and to regulate a variety of metabolic reactions.

GCN1 was the most interesting candidate we identified, because of its involvement in the regulation of protein synthesis during amino acid starvation in eukaryotes [9–12, 14, 15, 53, 58]. We validated this interaction between AaCAT1 and GCN1 by using in vitro pull-downs with His-tagged AaCAT1 bait and protein lysate prey. This does not preclude the possibility that another protein may bridge this interaction. To fully confirm this to be a direct interaction, in vitro pull-downs using purified AaCAT1 bait and GCN1 prey will need to be performed. However, our detection of this interaction through multiple assays gives us confidence that it is likely to occur in vivo regardless of whether the interaction is direct or bridged. In yeast, GCN1 forms a complex with several proteins, including the kinase, GCN2, and this complex binds to ribosomes where GCN2 can sense and become activated by uncharged tRNAs during amino acid starvation conditions [12–15]. Once activated, GCN2 phosphorylates the alpha subunit of the eukaryotic translation initiation factor-2 complex (eIF2α) [12–16], resulting in the downregulation of general translation, and the stimulation of translation of a small set of genes, including the transcription factor ATF4 (general control nonderepressible 4 [GCN4] in yeast), which regulate amino acid metabolism [20, 21, 59]. In addition, this signaling pathway has been implicated in regulation of mTOR activity in eukaryotes [23, 26]. Previous research has demonstrated that mTOR signaling is central during mosquito vitellogenesis [6, 31, 45, 55], and amino acid availability has been shown to affect mTOR and S6K activity in *Ae. aegypti* [30–32, 45].

Because the AaCAT1 interaction region is at the C-terminal end of GCN1 and does not overlap with ribosomal or GCN2 interaction sites in yeast [14, 60], we propose that GCN1 interacting with AaCAT1 can serve as a docking site for ribosomes. Interacting in this fashion would allow GCN1 to both rapidly sense changes in the amino acid state of the cell and increase the efficiency of YPP translation by recruiting ribosomes to areas of high amino acid concentration. It is also possible that interaction with GCN1 is necessary for AaCAT1 activation or maintenance of transporter stability during vitellogenesis, but our GCN1 knockdown data do not provide support for this notion. However, this possibility cannot be ruled out without further assays of AaCAT activity after GCN1 knockdown. The interaction that we observed between AaCAT1 and GCN1 can take place in several subcellular locations. First, GCN1 and AaCAT1 may interact at the plasma membrane of the cell. Second, GCN1 and AaCAT1 may interact at secretory vesicles as AaCAT1 is being transported to the cell membrane. Finally, GCN1 and AaCAT1 may interact at lysosomes. This location of interaction is possible, as AaCAT1 is predicted to localize either to the cell membrane or to lysosomes (Additional file 1: Figure S1), and lysosomes are known to serve as an amino acid storage site [61] and a hub for amino acid signaling by the TOR pathway [62, 63]. Further studies are required to elucidate which relationships may exist between GCN1, mTOR and lysosomes during vitellogenesis and where these interactions take place within the cell. To understand how GCN1 may intersect with mTOR signaling, a yeast two-hybrid study using GCN1 protein fragments as bait may identify interactions between GCN1 and proteins in the mTOR signaling pathway. Additionally, the advent of clustered regularly interspaced short palindromic repeat/CRISPR associated protein 9 (CRISPR/Cas9) gene editing and techniques such as receptor-mediated ovary transduction of cargo (REMOT Control), which allows the use of CRISPR/Cas9 to develop genetic lines by injecting adult female mosquitoes [64], makes the generation of GCN1 or mTOR signal pathway protein knockout lines feasible. This can allow for the design of experiments in a GCN1 or mTOR-signaling null genetic background which can further elucidate how GCN1 and the GAAC pathway may crosstalk with mTOR signaling to regulate vitellogenesis. CRISPR/Cas9-based editing can also take advantage of homology directed repair to precisely knock-in fluorescent labels into GCN1 or mTOR signaling genes, allowing for the use of fluorescent techniques, such as fluorescence resonance energy transfer (FRET), to identify if GCN1 directly interacts with mTOR or other mTOR signaling proteins in the *Ae. aegypti* fat body or other tissues at different times before and during vitellogenesis.

Western blot band densitometry of phosphorylated eIF2α normalized to eIF2α as a loading control showed a significant decrease in eIF2α phosphorylation 6 h PBM in mosquitoes injected with either GCN1 dsRNA or GFP dsRNA (Fig. 2d, e). This change is to be expected if the GAAC pathway is involved in nutrient sensation as female mosquitoes transition from a low amino acid diet of sucrose (in the laboratory) or nectar (in the wild) to a blood meal with a high amino acid content. We interpret this result to indicate that the GAAC pathway is playing an active role in mediating the metabolic switch to produce YPPs at the onset of vitellogenesis. When assaying for an effect of GCN1 knockdown on GAAC pathway activity, we did observe decreased eIF2α phosphorylation in GCN1 dsRNA-injected mosquitoes relative to GFP dsRNA-injected mosquitoes before the blood meal and in the first 24 h PBM (Fig. 2d, e). Statistical analysis of this decrease did not reveal a significant knockdown effect on eIF2α phosphorylation. GCN2 kinase activity has been shown to be regulated by the ribosomal P-stalk in other eukaryotes [65, 66], so it is possible that functional redundancy in GCN2 activation by mosquito ribosome P-stalks may explain why we observed non-significant reduction of eIF2α phosphorylation after knocking-down GCN1 expression.

Interestingly, while GCN1 knockdown did not affect the expression of *vg* mRNA as detected by qRT-PCR (Additional file 1: Fig. S3b), we did observe differences in the fecundity of female mosquitoes injected with GCN1 dsRNA relative to GFP dsRNA controls (Fig. 3). We have previously published fecundity and hatch rate data using sample sizes comparable to those were used in the present study [67], so while we are confident that our data accurately represent the effect of GCN1 knockdown on fecundity, future experiments designed to understand the precise role played by GCN1 in mosquito fecundity will include larger sample sizes. Knockdown of GCN1 should not inhibit other factors known to be necessary for YPP gene transcription, including 20-hydroxyecdysone (20-E) signaling, juvenile hormone signaling or insulin-like peptide signaling [31]. We propose three possible explanations for how GCN1 knockdown may affect clutch size without affecting YPP gene transcription in the experiments we performed. First, these results could be due to functional redundancy in GCN2 activation by ribosomal P-stalk proteins. Second, these results may be caused by increased protein translation in GCN1 dsRNA-treated mosquitos pre-blood meal which depleted pre-blood meal amino acid stores, causing reduced efficiency in YPP synthesis. Third, GCN1 knockdown may cause disordered ribosome recruitment during vitellogenesis which reduced efficiency of YPP synthesis. Future metabolomic and proteomic studies, and ribosome purification studies in GCN1 knockdown mosquitoes will provide insight into which if any of these mechanisms may play a role in GCN1-mediated change in fecundity.

Our analysis of GCN1 localization showed that GCN1 is expressed at higher levels in tissues involved in the processing of blood meal nutrients and vitellogenesis, particularly the ovaries, fat body and midgut. Interestingly, in the midgut of unfed female mosquitoes, we detected GCN1 mRNA with qRT-PCR, but did not detect GCN1 protein by western blot. We hypothesize that midgut tissues may regulate GCN1 protein expression in a different way than other metabolically important organs, such as by post-transcriptional regulation, as midgut cells do not store as many nutrients as the fat body and ovaries and may only require GCN1 activity at transient points around blood-feeding. Future studies should be designed to determine whether GCN1 mRNA is sequestered or targeted by microRNAs in this tissue. Our observation of increased GCN1 expression as detected by qRT-PCR and western blotting in ovaries relative to other adult female tissues (Fig. 2a) indicates that GCN1 plays an important role in ovary metabolism, which should be investigated. It is likely that GCN1 performs a similar function in both ovaries and fat body, as both tissues undergo significant metabolic changes during vitellogenesis. It may also be that endogenous GCN1 in the oocytes serves to regulate amino acid metabolism during embryogenesis. Recently, GCN1 has been implicated in regulating cell proliferation in a GCN2-independent fashion in mouse embryonic cells established from GCN1-mutant embryos [58]. Future studies, including direct determination of GCN1 knockdown effect on specific tissues, will need to be performed to determine the exact role played by the AaCAT1-GCN1 interaction in *Ae. aegypti* vitellogenesis.

## Conclusions

The identification of interactors of AaCAT1 and their involvement in cell signaling pathways provide important insight into nutrient signaling pathways. By identifying the proteins implicated in these pathways, chemical targets or inhibitors that are specific to the identified proteins may be designed. Our analysis of protein interactions with only one of five *Ae. aegypti* CATs has revealed a connection between AaCAT1 and a second nutrient sensor pathway that did not have a previously established role in *Ae. aegypti* nutrient signaling. With more CAT interactions to study, coupled with our expanding understanding of GCN1 activity, our picture of the nutrient sensor system in mosquitoes is only becoming more complex.

## Supplementary Information

**Additional file 1: Table S1.** Annotations of all CAT entries used for CAT annotation tree. **Table S2.** Potential protein interactors identified from AaCAT1 yeast two-hybrid screen. **Figure S1.** *Ae. aegypti* AaCAT1 is a transmembrane protein that localizes to the plasma membrane. **a.** Tertiary structure of the AaCAT1 protein was visualized using PyMOL (https://pymol.org/2/). **b.** The TMHMM transmembrane prediction tool, Krogh et al. [39] was used to predict the transmembrane domains, and intracellular and extracellular regions of AaCAT1 to design the bait fragment for yeast two-hybrid analysis. **c.** AaCAT1 is predicted to localize to the plasma membrane as determined by the DeepLoc-1.0 subcellular localization tool, Almagro Armenteros et al. [52]. **Figure S2.** In silico predictions of GCN1 subcellular localization. **a.** GCN1 is predicted to localize to the cytoplasm, but also may localize to the nucleus. **b.** Predicted nuclear localization sequences (NLS) found in GCN1 reveal several NLS with scores indicating a mixed nuclear-cytoplasmic localization pattern. **c.** Result from part b showing location of predicted NLS within the context of the GCN1 amino acid sequence. Subcellular localization prediction was performed using the DeepLoc-1.0 subcellular localization tool, Almagro Armenteros et al. [52]. NLS prediction was performed using the cNLS Mapper prediction tool, Kosugi et al. [54]. **Figure S3.** GCN1 knockdown is not lethal, and does not affect YPP transcription. **a.** Survival of GCN1 dsRNA-injected and GFP dsRNA-injected control mosquitoes was not significantly different up to five days post-injection. A log-rank Mantel-Cox test was used to test for significant differences between the two treatments. **b.** qRT-PCR analysis of *vg* transcription in GCN1 dsRNA-injected and GFP dsRNA-injected control mosquitoes. Three biological replicates were analyzed per treatment at each time point and *vg* mRNA levels were normalized to β-actin mRNA prior to calculating fold-change in *vg* expression through the first 24 hr PBM. Paired t-tests were used to test for significant differences between the two treatments at each time point. **Figure S4.** Western blot and Coomassie blue images used in main figures without cropping, color removal, and brightness/contrast correction. The label above each image contains a reference to the main text figure where the image is included.

**Additional file 2: Dataset S1.** Excel workbook containing all yeast two-hybrid prey sequences from colonies with single prey plasmid copies. Each row represents the result from a single colony and includes the sequence of prey inserts generated from the T7 promoter in the pGAD-T7 prey plasmid.

## Data Availability

The datasets supporting the conclusions of this article are available within the article (and its additional files) and from the corresponding author on reasonable request.

## Abbreviations

AaCAT1, 2, 3, 5: *Aedes aegypti* cationic amino acid transporter 1, 2, 3, 5, respectively
AAR: Amino acid response
ATF4: Activating transcription factor 4
Cas9: CRISPR associated protein 9
CAT: Cationic amino acid transporter
CAT1, 2, 4, 5: Cationic amino acid transporter 1, 2, 4, 5, respectively
cDNA: Complimentary DNA
CRISPR: Clustered regularly interspaced short palindromic repeat
DDO: Double drop out (media)
DMSO: Dimethyl sulfoxide
dNTP: Deoxyribonucleoside triphosphate
dsRNA: Double-stranded RNA
DSSO: Disuccinimidyl sulfoxide
DTT: Dithiothreitol
EDTA: Ethylenediaminetetraacetic acid
eIF2α: Eukaryotic translation initiation factor 2 subunit alpha
FRET: Fluorescence resonance energy transfer
GAAC: General amino acid control
GCN1, 2, 4, 20: General control nonderepressible 1, 2, 4, 20, respectively
GFP: Green fluorescent protein
HAT: Heterodimeric amino acid transporter
IGEPAL: Octylphenoxy poly(ethyleneoxy)ethanol, branched
MEGA: Molecular evolutionary genetics analysis
mRNA: Messenger RNA
mTOR: Mechanistic target of rapamycin
mTORC1: Mechanistic target of rapamycin complex 1
MYA: Million years ago
NCBI: National Center for Biotechnology Information
noRT: Non-reverse transcribed
oligo-dT: Oligomerized deoxythymidine
p70-S6K: Ribosomal protein S6 kinase beta-1
PBM: Post-blood meal
PI: Post-injection
PVDF: Polyvinylidene difluoride
QDO: Quadruple drop out (media)
qRT-PCR: Quantitative reverse transcription PCR
REMOT Control: Receptor-Mediated Ovary Transduction of Cargo
RNAi: RNA interference
rps7: Ribosomal protein S7
SD: Synthetic defined media
SDS-PAGE: Sodium dodecyl sulfate–polyacrylamide gel electrophoresis
SLC7: Solute carrier family 7
Slif: Slimfast
TAE: Tris-acetate EDTA buffer
TBS: Tris-buffered saline
TBST: Tris-buffered saline + 0.05% Tween-20
TOR: Target of rapamycin
tRNA: Transfer RNA
*vg*: Vitellogenin
YPP: Yolk precursor protein
20-E: 20-Hydroxyecdysone
6-His: 6-Histidine
