# Supplementary material for: General control nonderepressible 1 interacts with cationic amino acid transporter 1 and affects Aedes aegypti fecundity
Source: Parasit Vectors. 2022 Oct 21;15:383. doi: 10.1186/s13071-022-05461-x (PMC9587632; doi:10.1186/s13071-022-05461-x)

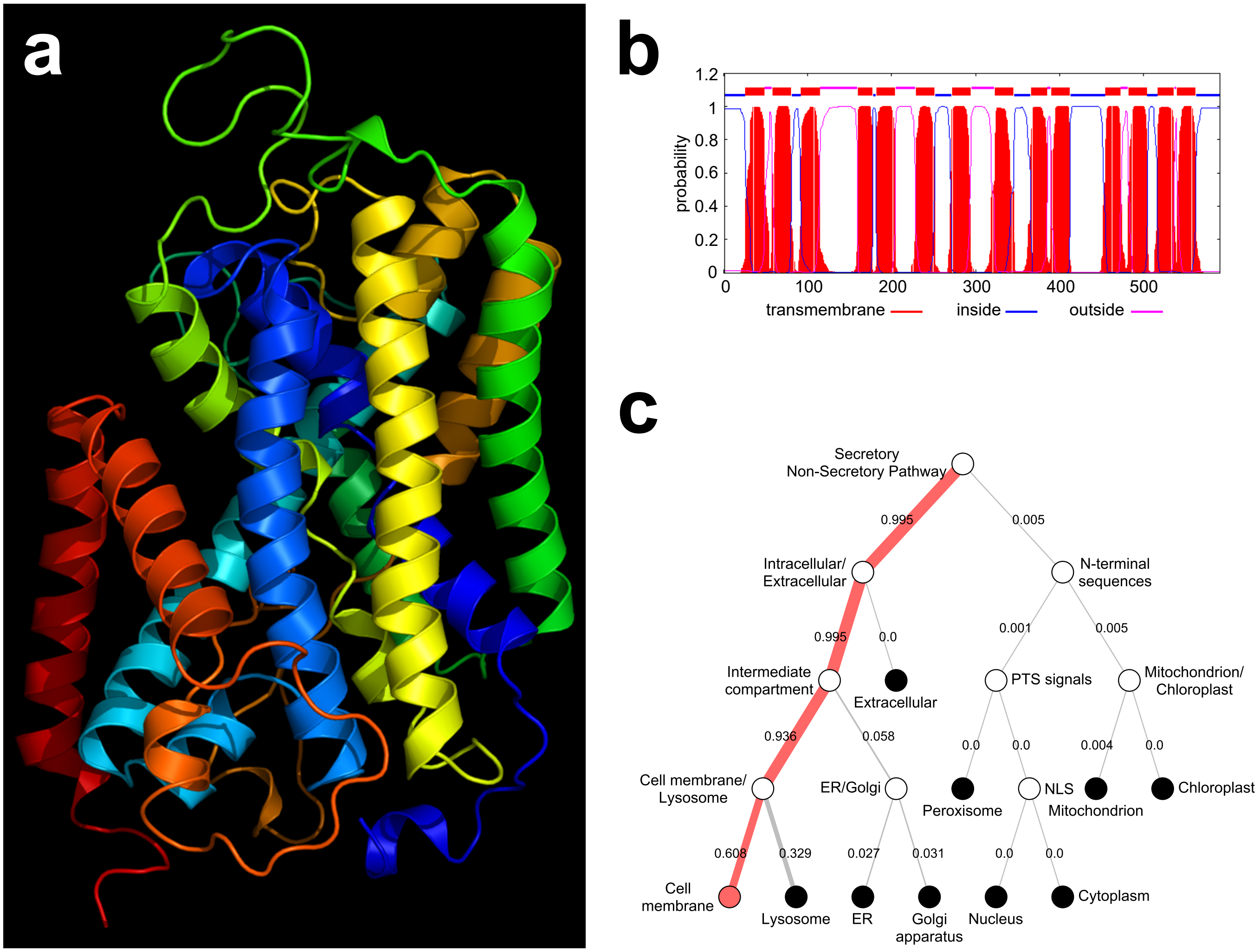

Figure S1. ***Ae. aegypti*** **AaCAT1 is a transmembrane protein that localizes to the plasma membrane.** **a.** Tertiary structure of the AaCAT1 protein was visualized using PyMOL (https://pymol.org/2/). **b.** The TMHMM transmembrane prediction tool, Krogh et al. [39] was used to predict the transmembrane domains, and intracellular and extracellular regions of AaCAT1 to design the bait fragment for yeast two-hybrid analysis. **c.** AaCAT1 is predicted to localize to the plasma membrane as determined by the DeepLoc-1.0 subcellular localization tool, Almagro Armenteros et al. [52].

Table S1. Annotations of all CAT entries used for CAT annotation tree.

Entrez ID	Annotation	Organism	Proposed re-annotation	Source
XP_001662273.1	AaCAT1	*Ae. aegypti*		Hansen et al. 2011, Carpenter et al. 2012
XP_001659995.1	AaCAT2	*Ae. aegypti*		Carpenter et al. 2012
XP_001662274.1	AaCAT3	*Ae. aegypti*		Carpenter et al. 2012
XP_001659990.1	AaCAT4	*Ae. aegypti*		Carpenter et al. 2012
XP_001661832.1	AaCAT5	*Ae. aegypti*		Carpenter et al. 2012
5575859	Cationic amino acid transporter 2	*Ae. aegypti*	Cationic amino acid transporter 1	
5571884	Cationic amino acid transporter 2	*Ae. aegypti*		
5575863	Cationic amino acid transporter 3	*Ae. aegypti*		
5575110	Cationic amino acid transporter 3	*Ae. aegypti*	Cationic Amino acid transporter 5	
5575860	Cationic amino acid transporter 4	*Ae. aegypti*		
AAT73699.1	Cationic amino acid transporter slimfast	*Ae. aegypti*		Attardo et al. 2006
AKN80692.1	Slimfast	*Ae. aegypti*		Boudko et al. 2015
AAZ07714.1	iCAT2	*Ae. aegypti*		Attardo et al. 2006
5568181	Probable cationic amino acid transporter	*Ae. aegypti*		
35370	Torn and diminished rhabdomeres	*D. melanogaster*		
40510	Slimfast	*D. melanogaster*		
39622	CG7255	*D. melanogaster*		
39990	CG5535	*D. melanogaster*		
40254	CG13248	*D. melanogaster*		
32986	CG12531	*D. melanogaster*		

Entrez gene IDs are provided for all current gene annotations, and protein IDs are provided for previous annotations. Proposed re-annotations are based on MEGA, Tamura et al. [38] multiple sequence alignment and neighbor-joining tree analysis presented in Fig. 1a.

Table S2. Potential protein interactors identified from AaCAT1 yeast two-hybrid screen.

Identified potential prey interactors	Number of hits	NCBI Accession number	Location in the cell
eIF-2-alpha kinase activator GCN1	3	XP_001651826.2	Cytoplasm/ Ribosome
Sodium/potassium-transporting ATPase subunit beta-2	3	XP_001654893.1	Plasma membrane
PREDICTED: Aedes aegypti long-chain-fatty-acid--CoA ligase 4 (LOC5574220), transcript variant X4, mRNA	2	XM_021849348.1	Peroxisome/ Mitochondria/ Plasma membrane/ Endoplasmic reticulum
Serine protease SP24D	2	XP_001659969.2	Cytoplasm/ Extracellular region
Protein dj-1beta	2	XP_001648396.2	Cytoplasm/ Mitochondria/ Nucleus
Serine/threonine-protein phosphatase 6 catalytic subunit	1	XP_001648846.1	Cytoplasm/ Mitochondria
14-3-3 protein zeta isoform X1	1	XP_001652301.1	Cytoplasm/ Endosome
PREDICTED: Aedes aegypti prostatic acid phosphatase (LOC5563717), transcript variant X3, mRNA	1	XM_021837530.1	Cytoplasm
Chymotrypsin-like protein	1	AAL85585.1	Cytoplasm
trypsin 3A1 isoform X1	1	XP_001652943.1	Cytoplasm
PREDICTED: Aedes aegypti serine protease easter (LOC5569890), mRNA	1	XM_001658734.3	Cytoplasm
Annexin B9 isoform X5	1	XP_021708898.1	Cytoplasm/ Endomembrane system
Guanine nucleotide-binding protein subunit beta-like protein	1	XP_001663282.1	Cytoplasm

a

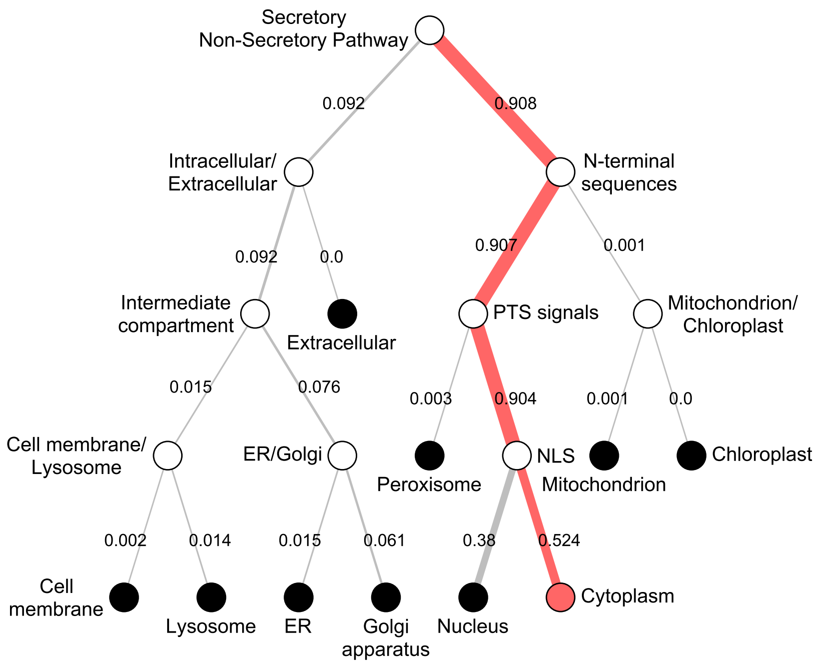

b

Predicted monopartite NLS		
**Pos.**	**Sequence**	**Score**
566	VNSPHKKVRKY	4.5
569	PHKKVRKYC	3

Predicted bipartite NLS		
**Pos.**	**Sequence**	**Score**
2608	REGDEVHQKCIALLEPGARDSLSEVVSKVLRKVAM	3.6

c

cNLS Mapper Result

Predicted NLSs in query sequence

```
MADAELGKALKDLPNRVLNVSLEERPELFRNVIDVLPNPGINATIVRGIC 50
KVIGTTLTKYKDPVSQGLVRELLVALLKQHPDLAYEHFNNVLKALVTKDL 100
AAAPPLKGAQAAVLALGWANLVALHGNHESDVGKKEFPKLIEIQAGLYQL 150
SLTSGIQKVSDKAYSFLKEFFAKKEDIDKVYFKKLIAMEPSSSGILLMSA 200
ILNYNKEEFEDHTLLDQNKAKLLDHVVKGLVTVKTKPHASDVTGCSILLS 250
AITKDEFKATILPALQRSMLRSPEVILRAVGAMVSEIPVDVSDFAMDLGK 300
TLVQNLASKDETVRQEAVESLKQVAMKCSAPKAVEALLKEVFAVFNGSGG 350
KITVVELRINLLQGAGNLSNNQIKPEDIVTIMPTVTDLFIKALETETQEK 400
VLCHALEMFGLWSVNFRGEIPTKIVQTFKKGLDVKAQVLRTSYLQWFLAC 450
LQNGKLPSGSDFTAPLSKIVEKAAQNPTQTPAVSEGVGAACIVLLTNPKV 500
SDQLKDFWNVVLDMNKKVFLGEKFLSATAPETLCYVMQFCERILLNHLDD 550
LKGSPEPLFKTVIHCVNSPHKKVRKYCLPLLKKIVNSPNGINLAKSLLLE 600
LTNFVENTKILNEGEQEEGVVPAQAIVDTVMSVTDIESIQISDAQTIALS 650
VLLCAHHPAPVSVQSDLWESILKRYELDGKYFISMNTSHIKEVFFNGYKA 700
TAMYENTLATLSHISPEVILPVLVKNVTDQLNNSKMSNVTDEEYFTYLTP 750
DGELYDKSVIPNSEEQTNTAHLKRENKAYSYKEQLEELTLRREIEEKRRK 800
EGKSKPPQLTPKQKEAIEKQTEKEKAIKTRLRELNSIITTLISQIEGAAK 850
GTPRELSLFFPTLLPAILRVFSSPLAAPSMVKLYLRLRETCFSGELVETG 900
RDVAIATIRLSKPHCDLEESWCTENLLELVSDILVSIYDETIDKYNVHIE 950
EDGAKNFLLNAPAFSYTFEFLKRAMVLPEAAKDESLLINGIQIIAYHAQI 1000
KGDTVDGQDFEDLYHPKYMPRLEMIKLLLNLIQHHRGRVQTQSVAALLDV 1050
AESSSGKEYTAKADTREVECLLVALQDELDAVRDVALRALAIMIKVLPSI 1100
SDDYELGLRLTRRLWVAKHDVCEDTKQLAENVWIQGNFEVPIVMADELLK 1150
DIIHPEPCIQKAASFALVSILAEDSSIIDSILEQLLEIYQDKLTMIPAKL 1200
DQFDREIEPAIDPWGPRRGVAIALSHVAPFLTEELVNSLTQFMVSTGLRD 1250
REEIVHKEMLAASLAIVEHHGKVSVSYLLPTFEKFLDEAPNNSDYDNIRQ 1300
AVVILMGSLARHLDRDDERIQPIVNRLLTALSTPSQQVQESVANCIPYLI 1350
PSVKDQAPQMVKKMMQQLVKSEKYGVRRGAAYGIAGIVKGLGILSLKQLD 1400
IMTKLTNYIQDKKNFKSREGALFAFEMLCSTLGRLFEPYIVHVLPHLLQC 1450
FGDSSSYVREAADECAKTVMAKLSAHGVKLILPSLLNALDEDSWRTKTAS 1500
VELLGAMAFCAPKQLSSCLPSIVPKLMEVLGDSHIKVQEAGATALKMIGS 1550
VIKNPEIQAIVPVLLRALENPSSKTSLCLQSLLETKFVHFIDAPSLALIM 1600
PVVQRAFMDRSTETRKMAAQIIGNMYSLTDQKDLTPYLPNIIPGLKTSLL 1650
DPVPEVRAVSARALGAMVRGMGESSFEDLLPWLMQTLTSESSSVDRSGAA 1700
QGLSEVVGGLGVEKLHKLMPEIIATAERTDIAPHVKDGYIMMFIYMPSAF 1750
PNDFTPYIGQIINPILKALADENEYVRDTALKAGQRIVNLYAESAIALLL 1800
PELEKGLFDDNWRIRYSSVQLLGDLLYKISGVSGKMTTQTASEDDNFGTE 1850
QSHKAIIRSLGAERRNRVLAGLYMGRSDVSLMVRQAALHVWKVVVTNTPR 1900
TLREILPTLFSLLLGCLASTSYDKRQVAARTLGDLVRKLGERVLPEIIPI 1950
LERGLNSDQADQRQGVCIGLSEIMASTSRDMVLTFVNSLVPTVRKALADP 2000
LPEVRQAAAKTFDSLHTTVGTRALDDILPSMLESLSDPDPDVAEWTLDGL 2050
RQVMAIKSRVVLPYLIPQLTANPVNTKALSILASVAGEALTKYLPKILPA 2100
LLSALAAAQGTPEEAQELEYCQAVILSVSDEVGIRTIMDTVMESTKSEIP 2150
ETRKAAATLLCAFCTHSPGDYSQYVPQLLRGLLRLLADGDRDVLQRSWDA 2200
LNAVTKTLDSAQQIAHVTDVRQAVKFASSDLKGAELPGFCLPKGITPLLP 2250
VFREAILNGLPEEKENAAQGLGEVIQLTSASSLQPSVVHITGPLIRILGD 2300
RFNAGVKAAVLETLAILLHKVGIMLKQFLPQLQTTFLKALHDPSRIVRIK 2350
AGHALAELIVIHTRPDPLFVEMQNGIKNTDDSTIRETMLQALRGILTPAG 2400
DKMTEPLKKQIYSMLSGMLGHPEDVTRAAAAGCLGALCRWLNPDQLDDAL 2450
NSHMLNEDYGDDAALRHGRTAALFVALKEYPSAIFTDKYEAKICKTIASS 2500
LISDKIPVALNGVRAAGYLLQYGMCSDDVKLPQQIIGPFVKSMNHTSNEV 2550
KQLLAKTCLYLAKTVPAEKTAPEYLRLVIPMLVNGTKEKNGYVKSNSEIA 2600
LVYVLRLREGDEVHQKCIALLEPGARDSLSEVVSKVLRKVAMQPVGKEEE 2650
LDDTILT 2657
```

Figure S2. ***In silico*** **predictions of GCN1 subcellular localization.** **a.** GCN1 is predicted to localize to the cytoplasm, but also may localize to the nucleus. **b.** Predicted nuclear localization sequences (NLS) found in GCN1 reveal several NLS with scores indicating a mixed nuclear-cytoplasmic localization pattern. **c.** Result from part b showing location of predicted NLS within the context of the GCN1 amino acid sequence.

Subcellular localization prediction was performed using the DeepLoc-1.0 subcellular localization tool, Almagro Armenteros et al. [52]. NLS prediction was performed using the cNLS Mapper prediction tool, Kosugi et al. [54].

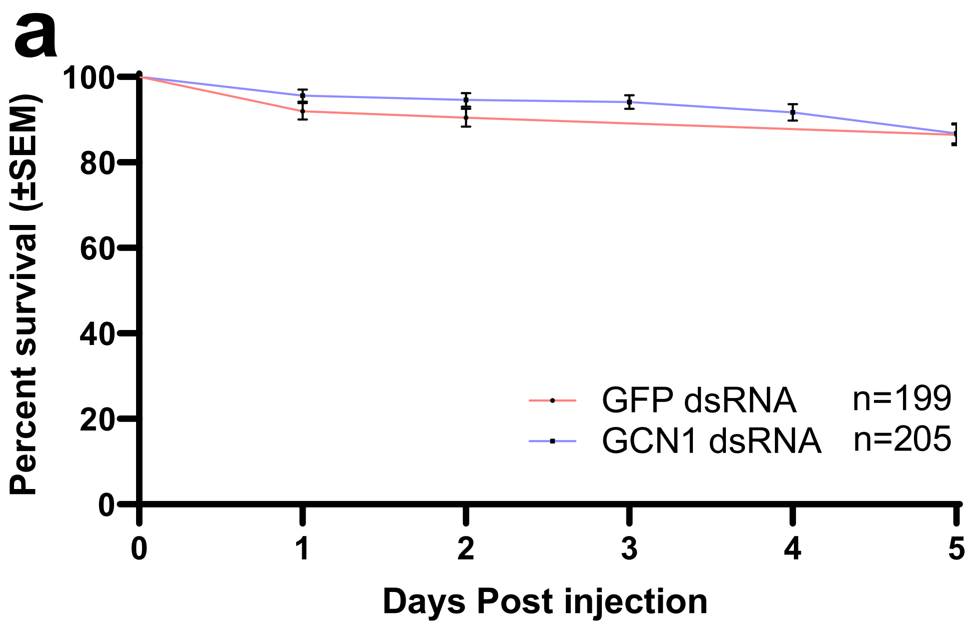

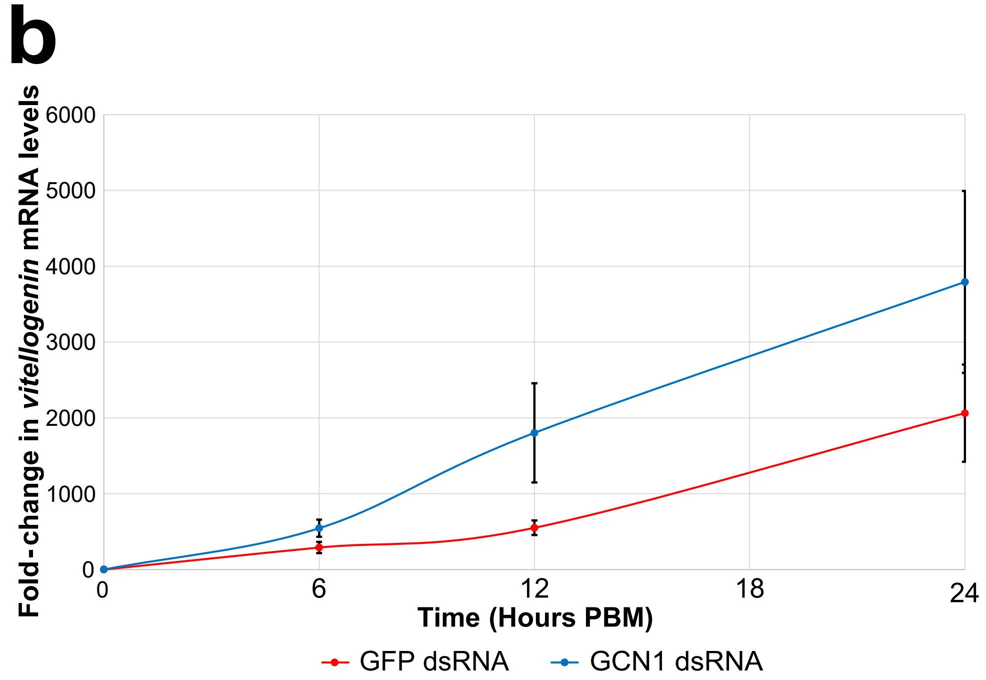

Figure S3. GCN1 knockdown is not lethal, and does not affect YPP transcription. a. Survival of GCN1 dsRNA-injected and GFP dsRNA-injected control mosquitoes was not significantly different up to five days post-injection. A log-rank Mantel-Cox test was used to test for significant differences between the two treatments. **b.** qRT-PCR analysis of *vg* transcription in GCN1 dsRNA-injected and GFP dsRNA-injected control mosquitoes. Three biological replicates were analyzed per treatment at each time point and *vg* mRNA levels were normalized to β-*actin* mRNA prior to calculating fold-change in *vg* expression through the first 24 hr PBM. Paired t-tests were used to test for significant differences between the two treatments at each time point.

Figure S4. The following pages contain Western blot and Coomassie blue images used in main figures without cropping, color removal, and brightness/contrast correction. The label above each image contains a reference to the main text figure where the image is included.

Figure S4a. Cross-link Coomassie stain gel (Fig. 1e).

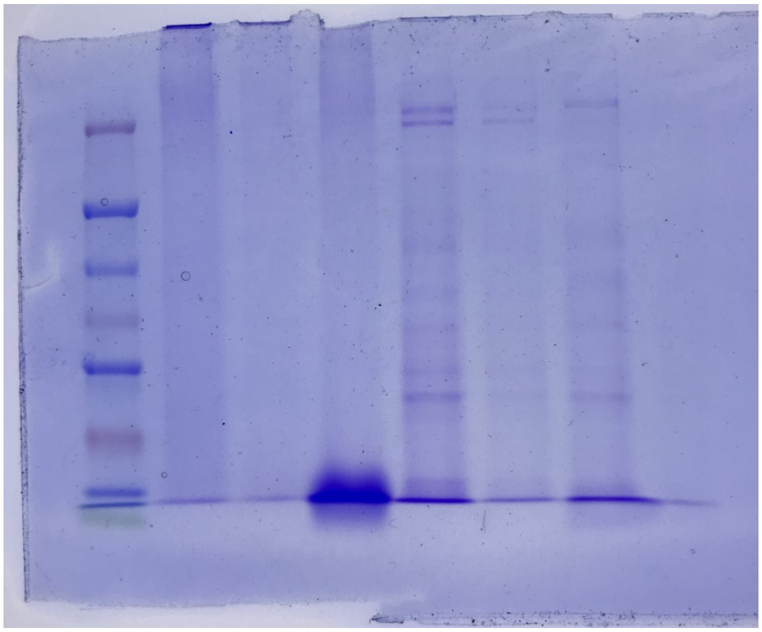

Figure S4b. Pulldown Western Blot (Fig. 1f).

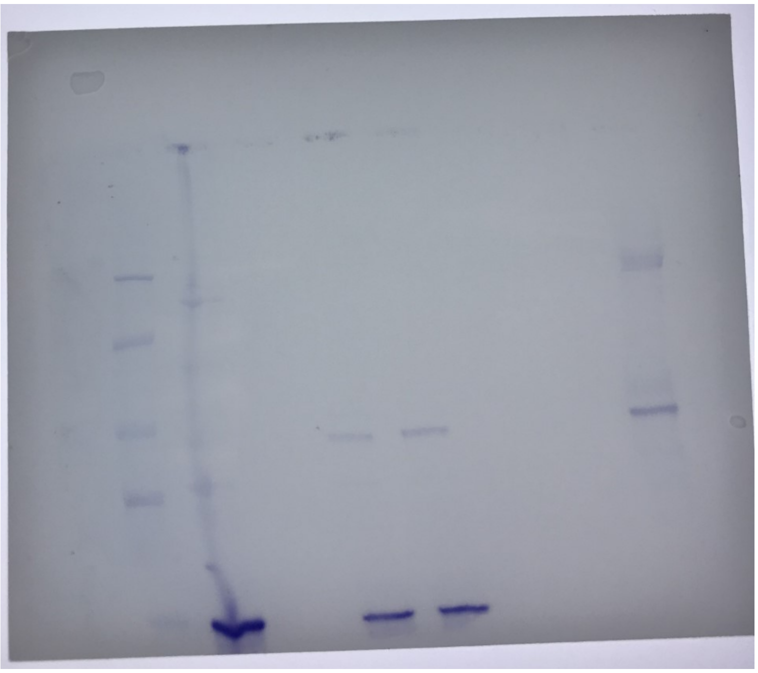

Figure S4c. GCN1 expression in adult tissues (Fig. 2a bottom).

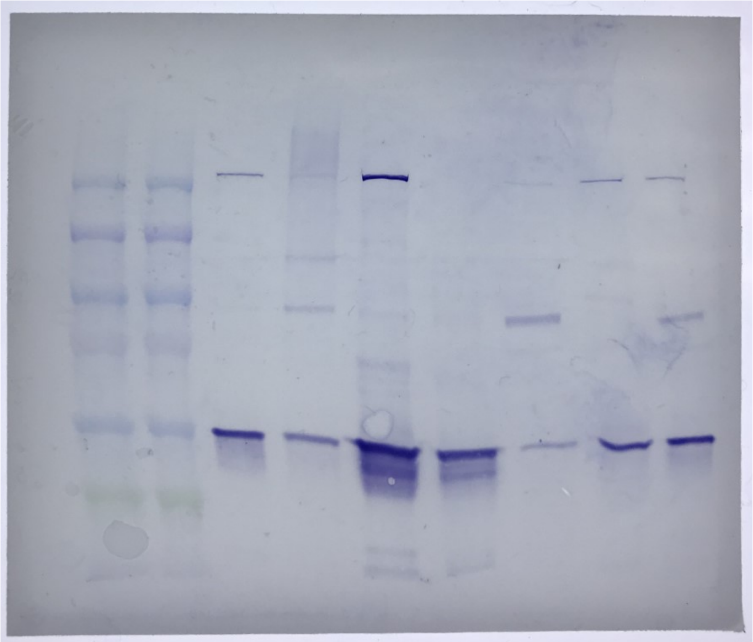

Figure S4d. Confirmation of GCN1 dsRNA knockdown (Fig. 2b).

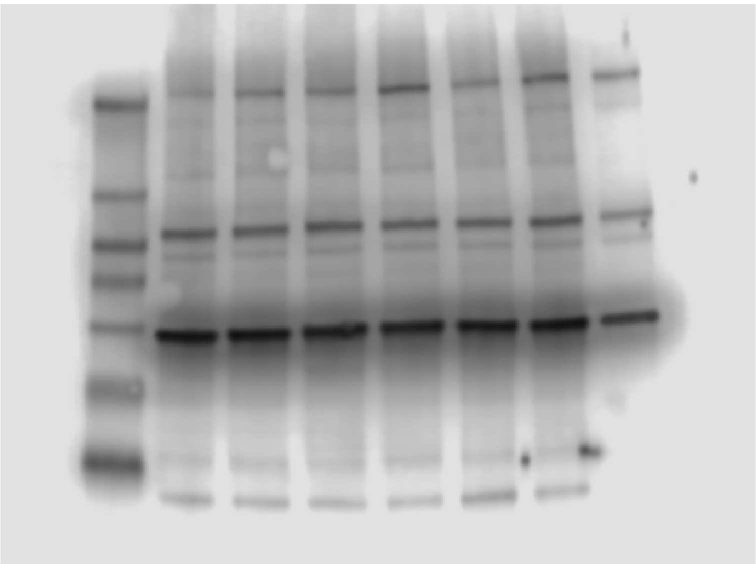

Figure S4e. Phospho-eIF2α Western blots (Fig. 2d top).

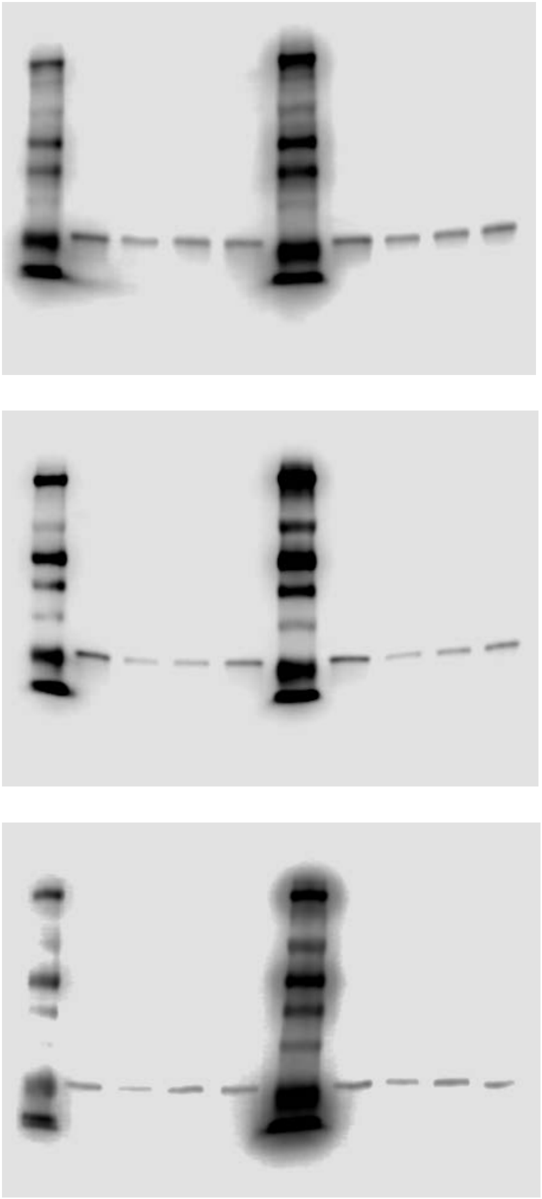

Figure S4f . eIF2α Western blots (Fig. 2d middle).

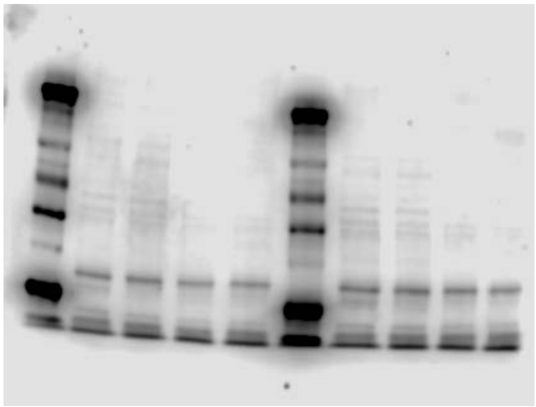

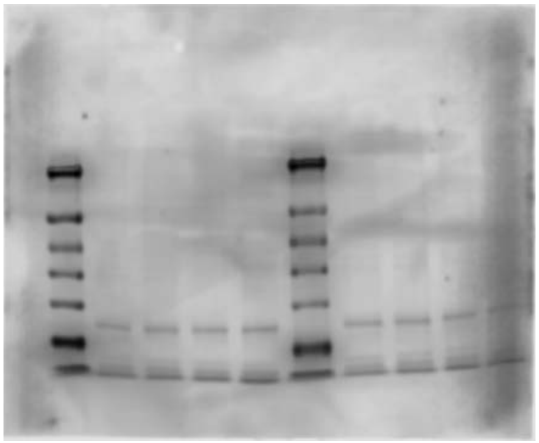

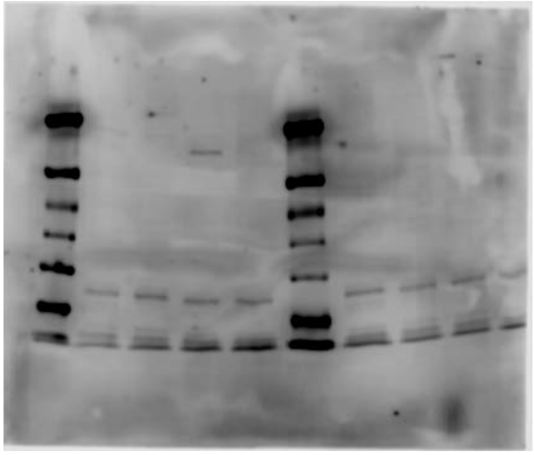

Figure S4g. Actin Western Blots
(Fig. 2d bottom).

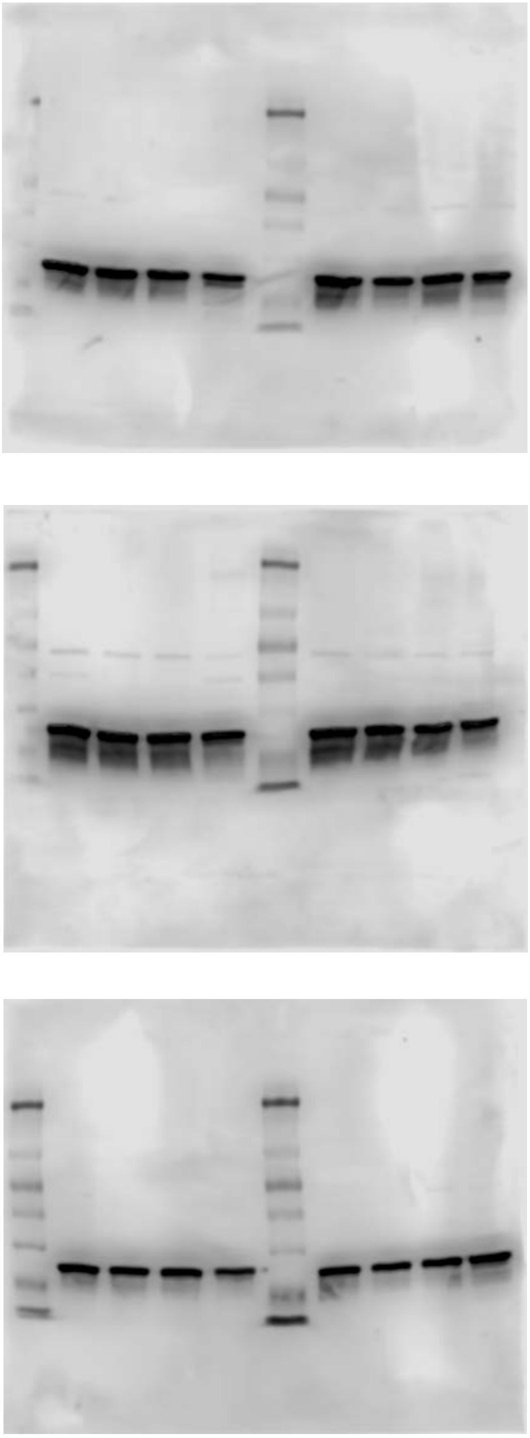

Supplement: Supplementary file 1 — Additional file 1: Table S1. Annotations of all CAT entries used for CAT annotation tree. Table S2. Potential protein interactors identified from AaCAT1 yeast two-hybrid screen. Figure S1. Ae. aegypti AaCAT1 is a transmembrane protein that localizes to the plasma membrane. a. Tertiary structure of the AaCAT1 protein was visualized using PyMOL (https://pymol.org/2/). b. The TMHMM transmembrane prediction tool, Krogh et al. [39] was used to predict the transmembrane domains, and intracellular and extracellular regions of AaCAT1 to design the bait fragment for yeast two-hybrid analysis. c. AaCAT1 is predicted to localize to the plasma membrane as determined by the DeepLoc-1.0 subcellular localization tool, Almagro Armenteros et al. [52]. Figure S2. In silico predictions of GCN1 subcellular localization. a. GCN1 is predicted to localize to the cytoplasm, but also may localize to the nucleus. b. Predicted nuclear localization sequences (NLS) found in GCN1 reveal several NLS with scores indicating a mixed nuclear-cytoplasmic localization pattern. c. Result from part b showing location of predicted NLS within the context of the GCN1 amino acid sequence. Subcellular localization prediction was performed using the DeepLoc-1.0 subcellular localization tool, Almagro Armenteros et al. [52]. NLS prediction was performed using the cNLS Mapper prediction tool, Kosugi et al. [54]. Figure S3. GCN1 knockdown is not lethal, and does not affect YPP transcription. a. Survival of GCN1 dsRNA-injected and GFP dsRNA-injected control mosquitoes was not significantly different up to five days post-injection. A log-rank Mantel-Cox test was used to test for significant differences between the two treatments. b. qRT-PCR analysis of vg transcription in GCN1 dsRNA-injected and GFP dsRNA-injected control mosquitoes. Three biological replicates were analyzed per treatment at each time point and vg mRNA levels were normalized to β-actin mRNA prior to calculating fold-change in vg expression through t [file 13071_2022_5461_MOESM1_ESM.pdf]
